# Structure-efficiency relationship of photoinduced electron transfer-triggered nitric oxide releasers

**DOI:** 10.1038/s41598-018-38252-5

**Published:** 2019-02-05

**Authors:** Naoya Ieda, Yumina Oka, Toshitada Yoshihara, Seiji Tobita, Takahiro Sasamori, Mitsuyasu Kawaguchi, Hidehiko Nakagawa

**Affiliations:** 1 Graduate School of Pharmaceutical Sciences, Nagoya City University, 3-1, Tanabe-dori, Mizuho-ku, Nagoya, Aichi 467-8603 Japan; 2 Faculty of Pharmaceutical Sciences, Nagoya City University, 3-1, Tanabe-dori, Mizuho-ku, Nagoya, Aichi 467-8603 Japan; 3 Graduate School of Science and Technology, Gunma University, 1-5-1, Tenjin-cho, Kiryu, Gunma, 376-8515 Japan; 4 Graduate School of Natural Sciences, Nagoya City University, 1, Yamanohata, Mizuho-cho, Mizuho-ku, Nagoya, Aichi 467-8501 Japan

## Abstract

Spatiotemporally controllable nitric oxide (NO) releasers are required for biological studies and as candidate therapeutic agents. Here, we investigate the structure-efficiency relationship of a series of photoinduced electron transfer-triggered NO releasers based on our reported yellowish-green light-controllable NO releaser, NO-Rosa. The distance between the NO-releasing *N*-nitrosoaminophenol moiety and the rosamine antenna moiety was critical for efficient NO release. Notably, substitution at the phenolic hydroxyl group blocked NO release. We synthesized NO-Rosa-Gal bearing D-galactose (Gal) at this location, and showed that hydrolysis by β-galactosidase restored the photoresponse. This represents proof-of-concept of a strategy for highly specific control of NO release by using a double-lock system involving both enzymatic reactivation and photo-control.

## Introduction

Nitric oxide (NO) is an endogenous regulator of various physiological events, including vasodilation, immune response, and neural transmission^1–4^. However, it is very difficult to handle NO in biological experiments because it is a low-molecular-weight, gaseous, reactive compound under ambient conditions. Therefore, various NO releasers have been developed as chemical tools for NO research and as chemotherapeutic agents to treat heart disease^5^. Among them, photocontrollable NO releasers are expected to be most useful as chemical tools and phototherapeutic reagents, because they offer very high spatiotemporal controllability of NO release^6–12^.

**Figure 1:**
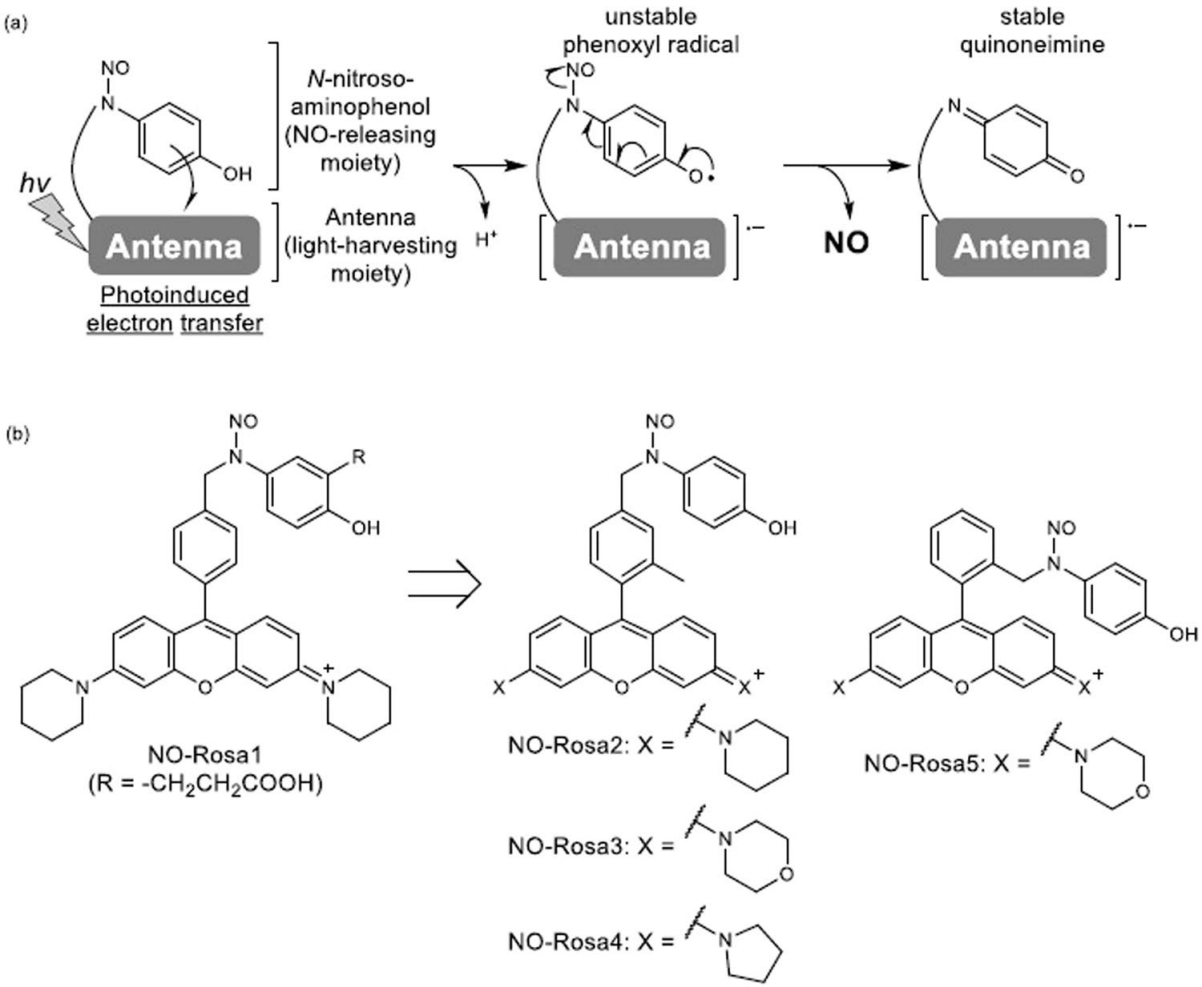
A plausible mechanism of PeT triggered NO releasers (**a**) and structure of NO-Rosa1–5 (**b**).

We previously developed intramolecular photoinduced electron transfer (PeT)-triggered NO releasers consisting of *N*-nitrosoaminophenol as the NO-releasing moiety and a visible-light-harvesting dye as the antenna moiety (Fig. 1a). Photo-excitation of the antenna moiety results in PeT to form the phenoxyl radical of the NO-releasing moiety and the anion radical of the antenna moiety. This phenoxyl radical is unstable, and the *N*-*N* bond is cleaved homolytically to release relatively stable NO and quinoneimine^13^. We utilized this mechanism to develop a blue-light-controllable NO releaser, NOBL-1, and a yellowish-green-light-controllable one, NO-Rosa1 (“NO-Rosa” in the previous report)^14^. However, NO-Rosa1 was difficult to synthesize efficiently due to its high polarity and instability. Therefore, the aim of the present work was to synthesize a series of NO-Rosa1 derivatives in order to investigate the structure-efficiency relationship of PeT-triggered NO release in response to yellowish-green light irradiation. The results indicated that π-π stacking between the NO-releasing moiety and the antenna moiety significantly improves the NO-releasing efficiency. Further, we found that *O*-alkylation of the NO-releasing moiety blocked NO release by changing the photodecomposition pathway. We further utilized these findings to develop a double-lock system employing both enzymatic reactivation and photo-control to intend highly specific regulation of NO release.

## Results and Discussion

In order to investigate the structure-efficiency relationship of PeT-triggered NO release, we first synthesized NO-Rosa2, 3, and 4, because Lavis’ group reported that the substituent groups in these derivatives affect the excited state of the fluorophore (Fig. 1b)^15^. Further, NO-Rosa5 was designed to alter the distance between the NO-releasing moiety and the antenna moiety, since a proximity effect can improve the efficiency of charge transfer^16,17^.

Although NO-Rosa1 has a carboxy group on the NO-releasing moiety^13^, we omitted this group in NO-Rosa2–5, since its high polarity made synthesis and purification difficult (Scheme S1). Furthermore, in the synthesis of NO-Rosa1, we found that the (4-hydroxyphenyl)amino group of S4, a precursor of the NO-releasing moiety, was readily oxidized by NaNO_2_ in the *N*-nitrosylation step, generating a large amount of aldehyde S1 as a by-product. To avoid this, we protected the phenolic hydroxyl group with a *tert*-butyldimethylsilyl (TBS) group, which could be deprotected under mild conditions with NaF/HF buffer in the final step (Schemes S2, S3)^18^. Nevertheless, NO-Rosa2–5 were obtained in better yields than NO-Rosa1. The structures of NO-Rosa2–5 were confirmed by means of ^1^H NMR, ^13^C NMR, ESI-MS, and elemental analyses.

**Figure 2:**
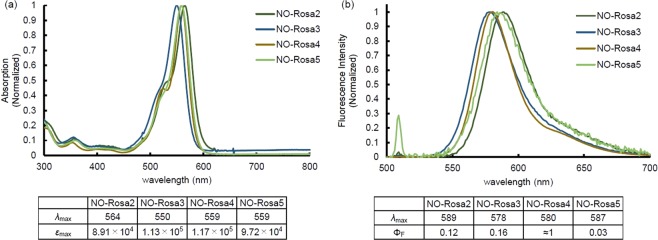
Absorption and fluorescence spectra of NO-Rosa2–5. (**a**) Absorption spectra of sample solutions (10 µM of each compound) in 100 mM HEPES buffer (pH 7.3, DMSO 0.1%); (**b**) Fluorescence spectra of sample solutions (10 µM of each compound) in 100 mM HEPES buffer (pH 7.3, DMSO 0.1%). Ex: 510 nm, both band widths were 3 nm.

**Figure 3:**
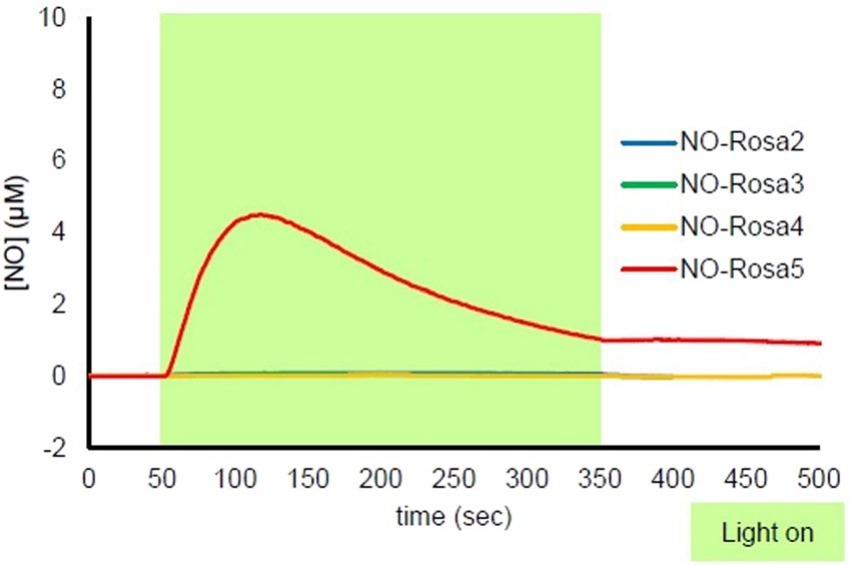
Measurement of NO release from NO-Rosa2–5 with an ISO-NOP NO electrode. Sample solutions of 10 µM test compound in 100 mM HEPES buffer (pH 7.3, DMSO 0.1%) were irradiated with a MAX-303 (Asahi Spectra) equipped with a 530–590 nm band-pass filter (light intensity: 70 mW/cm^2^).

The absorption spectra of NO-Rosa2–5 are shown in Fig. 2a. Since each compound exhibited strong absorption at around 560 nm, we used a Xe lamp, MAX-303 (Asahi Spectra) with a 530–590 nm band-pass filter for NO release experiments. The NO release during photoirradiation was monitored with an ISO-NOP NO electrode (World Precision Instruments). As shown in Fig. 3, NO-Rosa2–4 did not show efficient NO release in response to yellowish-green light irradiation, whereas NO-Rosa5 released NO efficiently. NO-Rosa5 released NO only during light irradiation, and the NO release was precisely responsive to light irradiation (Fig. S1). The maximum concentration of NO from 10 µM of NO-Rosa5 was reached to 4.5 µM. Also, we quantified the maximum [NO_2_^−^ + NO_3_^−^] by using NO_2_/ NO_3_ Assay Kit-FX (Dojindo, Japan). It is known that NO is readily oxidized under air to form NO_2_^−^ and NO_3_^−^. After NO-Rosa5 (10 µM) was completely photodecomposed, the amount of [NO_2_^−^ + NO_3_^−^] was determined as 6.6 µM.

**Figure 4:**
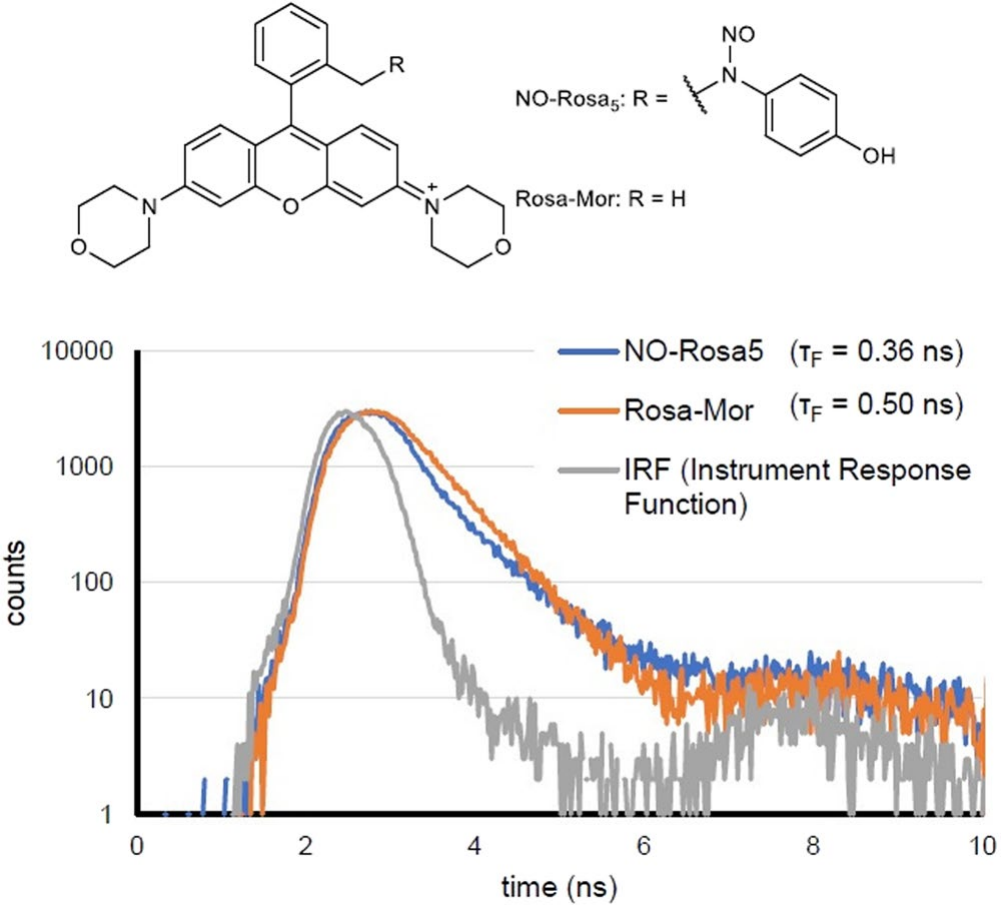
Fluorescence decay curves of NO-Rosa5 and Rosa-Mor.

To evaluate the results in more detail, we prepared Rosa-Pip, Rosa-Mor, and Rosa-Pyr as reference compounds without the NO-releasing moiety (Fig. S2)^19^. In addition to the absorption and fluorescence spectra, the time-resolved fluorescence was measured to determine the fluorescence lifetime (τ_F_) (Figs 2 and S3, S4). As shown in Figs 2a and S3a, the absorption spectra of NO-Rosa2–4 were almost the same as those of the reference compounds, whereas that of NO-Rosa5 was red-shifted by about 10 nm in comparison with that of Rosa-Mor, presumably as a result of π-π stacking of the NO-releasing moiety and antenna moiety^20^. In accordance with Lavis’ report, Rosa-Pyr showed the highest value of fluorescence quantum yield (Φ_F_), and the longest τ_F_ among these reference compounds (Figs S3b, S4c)^15^. If PeT from the NO-releasing moiety to the antenna moiety takes place efficiently, NO-Rosa derivatives should show lower Φ_F_ and shorter τ_F_ than those of reference compounds. But, in the case of NO-Rosa2–4, their Φ_F_ and τ_F_ were almost the same as those of the reference compounds (Figs 2b and S3b, S4a–c). Interestingly, NO-Rosa5 showed a biexponential decay curve in time-resolved fluorescence measurement, indicating that there were two components in the excited state (Fig. 4). Analysis of the fluorescence decay curve indicated that the ratio of these two components was 94:6. The fluorescence lifetime of the major component was 0.32 ns, while that of the minor one was 1.02 ns. From these results, the average fluorescence lifetime of NO-Rosa5 was estimated to be 0.36 ns (Table S1), whereas that of Rosa-Mor was 0.50 ns. Also, NO-Rosa5 showed a lower Φ_F_ than that of Rosa-Mor (NO-Rosa5: 0.03, Rosa-Mor: 0.16, Figs 2b, 4 and S3b). These results indicate that PeT does not take place in NO-Rosa2–4, whereas PeT and the subsequent NO release proceed efficiently in NO-Rosa5.

A possible explanation of the biexponential decay curve of NO-Rosa5 would be the existence of two conformational isomers (Fig. S5), of which one would be stacked and one would not in the excited state. PeT would proceed more efficiently in the stacked form than in the unstacked form. The major component is likely to be the stacked form, because its fluorescence lifetime should be shorter than that of the unstacked form due to more efficient electron transfer.

To confirm the spontaneity of PeT, the Gibbs free energy (*ΔG*_PeT_) was calculated according to Equation(1)^21^.

1 $${\rm{\Delta }}{G}_{PeT}=F\{{E}_{{\rm{o}}{\rm{x}}}(D){\textstyle \text{-}}{E}_{{\rm{r}}{\rm{e}}{\rm{d}}}(A)\}{\textstyle \text{-}}{E}_{0;0}+C$$

where *F* is the Faraday constant; *E*_*ox*_*(D)* is the oxidation potential of the electron donor, *E*_*red*_*(A)* is the reduction potential of the electron acceptor; *E*_0;0_ is the zero vibrational electronic excitation energy of the fluorophore, calculated as the average energy of the absorption and emission wavelengths of the fluorescence transition (2.21 eV in Rosa-Mor); and *C* is a term accounting for Coulombic interactions, which are typically assumed to be negligible in water. To calculate *ΔG*_*PeT*_ of NO-Rosa5 according to Equation(1), we measured the reduction potential of Rosa-Mor (*E*_red_ (A)) and the oxidation potential of *N*-methyl-*N*-nitroso-4-aminophenol (S26, *E*_*ox*_*(D)*), which corresponds to the NO-releasing moiety, by cyclic voltammetry and differential pulse voltammetry (Fig. S6). Indeed, *E*_*ox*_*(D)* and *E*_*red*_*(A)* were 0.97 V and –0.60 V (vs SCE), respectively, and *ΔG*_*PeT*_ was calculated to be –0.64 eV. This value is consistent with the observation that PeT between the NO-releasing moiety and the antenna moiety takes place in NO-Rosa5.

To quantify the NO-releasing efficiency of NO-Rosa5 in response to a yellowish-green light source, we determined the quantum yield of NO release (Φ_NO_) using a potassium ferrioxalate actinometer and an NO electrode^22^. The Φ_NO_ of NO-Rosa5 was determined as 1.01 × 10^–3^. The photolytic efficiency, *ε*_559_ _nm_ · Φ_NO_ was calculated to be 98.2, which is greater than the values reported for *o*-nitrobenzyl ester caged NOs (*ε* · Φ = 15–70)^6^.

**Figure 5:**
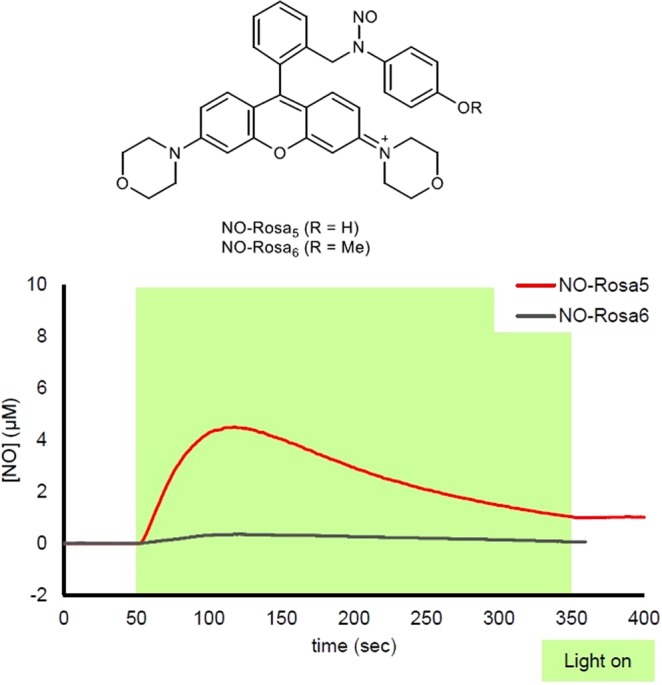
Measurement of NO release from NO-Rosa5 and 6 with the ISO-NOP. Sample solutions of each compound (10 µM) in 100 mM HEPES buffer (pH 7.3, DMSO 0.1%) was irradiated with a MAX-303 (Asahi Spectra) equipped with a 530–590 nm band-pass filter (light intensity: 70 mW/cm^2^).

In terms of redox potential, phenol is more readily subject to one-electron oxidation than anisole (phenol: 1.57 V, anisole: 2.00 V vs SHE)^23^. Therefore, if the phenolic hydroxyl group on the NO-releasing moiety were alkylated, PeT and the subsequent NO release should be suppressed. This would be consistent with the idea that the efficient NO release is due to one-electron oxidation of the moiety. To confirm this hypothesis, we synthesized and evaluated NO-Rosa6, which is an *O*-methylated form of NO-Rosa5 (Fig. 5 and Scheme S4).

As shown in Fig. 5, the NO-releasing efficiency of NO-Rosa6 was clearly less than that of NO-Rosa5. This result initially suggested that *O*-alkylation of the NO-releasing moiety simply suppressed PeT and subsequent photodecomposition. However, it turned out that the color quenching was faster than that of NO-Rosa5 (Fig. S7a,b), and the irradiated solution of NO-Rosa6 showed no color. Interestingly, this colorless solution acquired a pale red color after addition of a drop of aqueous HCl (Fig. S7c). To elucidate the reaction of NO-Rosa6, we conducted LC-MS analysis of irradiated solutions of NO-Rosa5 and NO-Rosa6 (Fig. S8). In an irradiated solution of NO-Rosa5, aldehyde derivative S22 was observed as the main photodecomposition product. On the other hand, an irradiated solution of NO-Rosa6 contained less S22, and also contained denitrosylated compound S25 as another photodecomposition product. Further, NO-Rosa6 showed a shorter fluorescence lifetime than Rosa-Mor, implying that PeT can take place even in NO-Rosa6 (Fig. S9). Taking these experimental results together, it was indicated that NO-Rosa6 decomposed via an alternative pathway not involving release of NO, but still via photoinduced electron transfer. Presumably, after PeT in NO-Rosa6, nitrosonium cation (NO^+^), which is equivalent to nitrite anion (NO_2_^−^) in neutral buffer solution, would be released, in contrast to the case of NO-Rosa5, because the charge-transferred intermediate could not undergo deprotonation (Scheme S5). After NO^+^ release followed by formation of NO_2_^–^, the denitrosylated compound S25 would be isomerized to a colorless spirocyclized product S27, which could revert to S25 under acidic conditions. Although it has been reported that NO_2_^−^ shows NO-like activity under biological conditions, it is less reactive than NO because reduction or an acidic condition is necessary for it to be biologically activated^24^.

**Figure 6:**
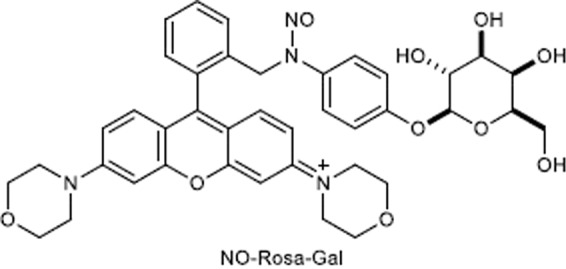
Structure of NO-Rosa-Gal.

Since *O*-methylation in NO-Rosa5 suppressed NO release by changing the decomposition pathway, we expected that *O*-alkylation could also be utilized as another regulatory factor to control NO release. Namely, we considered that *O*-alkylated NO-Rosa5, whose alkyl group could be cleaved by a specific enzyme, would serve as a double-locked NO releaser, which could be unlocked in living tissue by the combination of a specific enzymatic activity and light irradiation. To test this idea, and for proof of concept, we designed and synthesized NO-Rosa-Gal, whose hydroxy group is protected by D-galactose, which would be hydrolytically released by β-galactosidase (Fig. 6 and Scheme S6)^25^.

**Figure 7:**
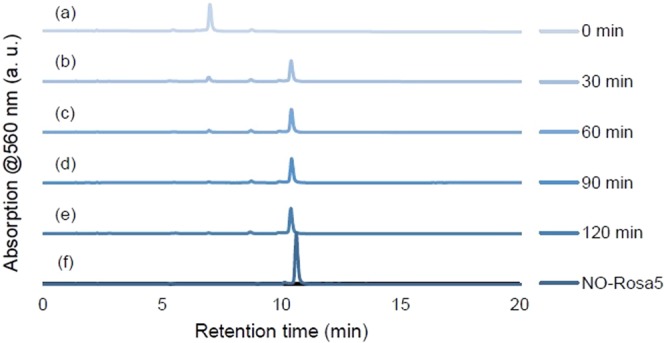
Enzymatic hydrolysis of NO-Rosa-Gal monitored by HPLC. A solution of NO-Rosa-Gal (10 µM) and β-galactosidase (10 U/mL) in HBSS (0.1% DMSO) was incubated at 37 °C for 0 min (**a**), 30 min (**b**), 60 min (**c**), 90 min (**d**), and 120 min (**e**). Chromatogram (**f**) shows authentic NO-Rosa5 (10 µM).

The hydrolysis of NO-Rosa-Gal by β-galactosidase was monitored by HPLC. As shown in Fig. 7, the hydrolysis of NO-Rosa-Gal (10 µM) was completed within one hour upon incubation with β-galactosidase (10 U/mL) in Hank’s balanced salt solution (HBSS) at 37 °C, while hydrolysis did not proceed in the absence of β-galactosidase (Fig. S10).

**Figure 8:**
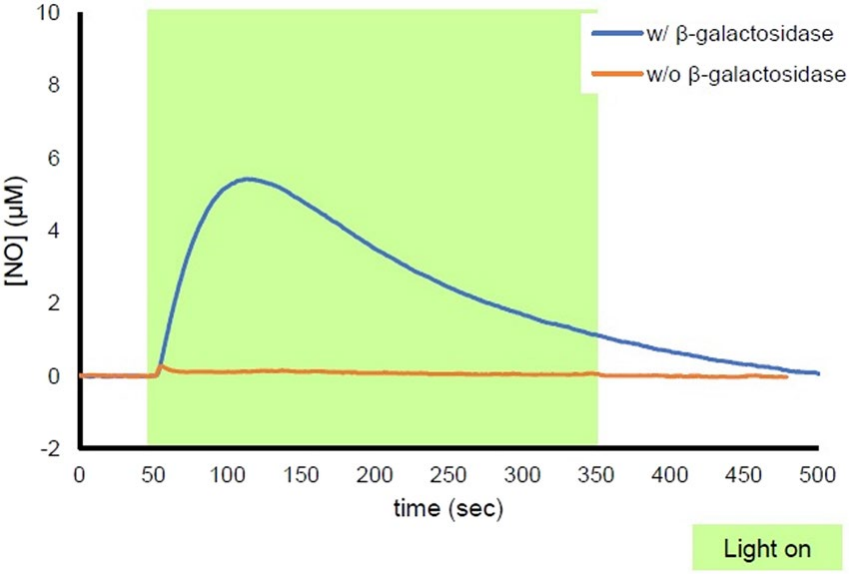
Measurement of NO release from NO-Rosa-Gal by ISO-NOP. A solution of NO-Rosa-Gal (10 µM) in HBSS (0.1% DMSO) was incubated in the presence or absence of β-galactosidase (10 U/mL). After incubation for one hour at 37 °C, the solution was irradiated with a MAX-303 (Asahi Spectra) equipped with a 530–590 nm band-pass filter (light intensity: 120 mW/cm^2^).

**Figure 9:**
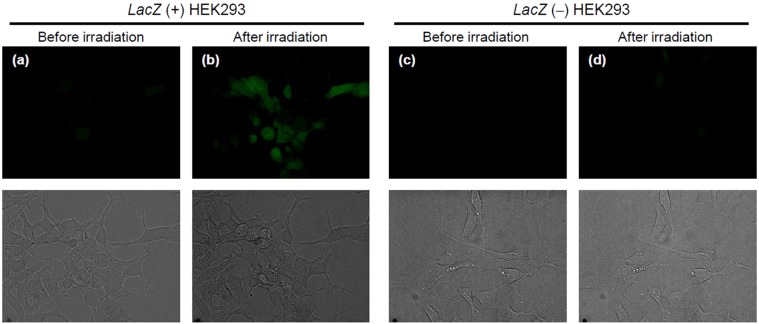
Fluorescence imaging of NO release from NO-Rosa-Gal in *lacZ*-HEK293 cells using DAF-FM DA. Cultured HEK293 cells were treated with DAF-FM DA (10 μM) and NO-Rosa-Gal (10 μM). The dishes were then photoirradiated with blue light (530−590 nm, 84 mW/cm^2^ for 15 min). The dishes were observed with a confocal fluorescence microscope. Upper figures are green fluorescence, and bottom figures are bright filed. (**a**) before photoirradiation to *lacZ* (+) cells, (**b**) after photoirradiation to *lacZ* (+) cells, (**c**) before photoirradiation to *lacZ* (−) cells, (**d**) after photoirradiation to *lacZ* (−) cells.

Finally, we examined the feasibility of dual enzymatic and photochemical control of NO release from NO-Rosa-Gal. After incubation of NO-Rosa-Gal (10 µM) in the presence or absence of β-galactosidase (10 U/mL), the solution was irradiated with a Xe lamp (MAX-303) equipped with a 530–590 nm band-pass filter. As shown in Fig. 8, in the absence of β-galactosidase, photoirradiation did not trigger NO release, whereas photoresponsive NO release was observed in the presence of β-galactosidase. These results indicated that *O*-alkylated PeT-triggered NO releasers could indeed mediate highly specific NO release under the dual control of enzymatic activity and photoirradiation. Also, NO-Rosa-Gal was applied for *lacZ* (+) HEK293 cells. *LacZ* reporter which encodes *Escherichia coli* β-galactosidase is a powerful reporter in combination with synthetic substrates^26^. To monitor NO release in cellular condition, the cells were treated with DAF-FM DA, a green fluorescence NO probe^27^. As shown in Fig. 9, fluorescence increment was observed in *lacZ* (+) HEK293 cells after yellowish-green light irradiation whereas the fluorescence increment in *lacZ* (−) HEK293 cells was less than that of *lacZ* (+) HEK293 cells. Also, in the absence of NO-Rosa-Gal, the fluorescence increment was not observed at all (Fig. S11). This in turn implies that *O*-protection strategy in PeT-driven NO releasers could be used to target NO release with great precision to cells overexpressing a specific enzyme.

## Conclusion

Spectroscopic studies indicated that a proximity effect, probably through π-π stacking, could significantly improve the NO-releasing efficiency of our PeT-triggered NO releasers in response to yellowish-green photoirradiation. On the other hand, *O*-alkylation of these PeT-triggered NO releasers blocked NO release by changing the decomposition pathway. These results will be useful for the design of improved NO releasers in the future. Further, we considered that they could provide a means of achieving highly specific, targeted NO release via a dual activation strategy. To test this idea, we examined the effect of substitution with D-galactose (NO-Rosa-Gal). Indeed, the photoresponse was blocked by the Gal substituent, but hydrolysis with β-galactosidase restored the response, opening the way for highly specific control of NO release by means of a double-lock system involving both enzymatic reactivation and photo-control. This strategy might be useful for the highly specific delivery of NO to cells overexpressing a targeting enzyme.

## Materials and Methods

Proton nuclear magnetic resonance spectra (^1^H NMR) and carbon nuclear magnetic resonance spectra (^13^C NMR) were recorded on a JEOL JNM-LA500, JEOL JNM-A500, Varian VNMRS 500, or Bruker AVANCE600 spectrometer in the indicated solvent. Chemical shifts (δ) are reported in parts per million relative to the internal standard, tetramethylsilane. Elemental analysis was performed with Yanaco CHN CORDER NT-5 analyzer, and all values were within ±0.4% of the calculated values. Ultraviolet-visible-light absorption spectra were recorded on an Agilent 8453 spectrometer. Fluorescence spectra were recorded on RF-5300 PC (Shimadzu). Irradiation was conducted with Asahi Spectra irradiating apparatus (MAX-303). All other reagents and solvents were purchased from Sigma-Aldrich, Tokyo Kasei Kogyo, FUJIFILM Wako Pure Chemical Corp., Nacalai Tesque, Kanto Kagaku, Kishida Kagaku, Junsei Kagaku or Dojindo, and used without purification. β-D-Galactosidase was purchased from FUJIFILM Wako Chemical Corp. (cat# 072-04141). Flash column chromatography was performed using silica gel 60 supplied by Taiko Shoji. MPLC purification was performed using YFLC-Wprep2XY-S (Yamazen).

Other details regarding synthesis, NMR chart, absorbance and fluorescence spectra, measurement of quantum yields, time-resolved fluorescence measurement, NO electrode, electrochemical measurement, LC-MS, HPLC, and cell culture are available in Supplementary Materials and Methods.

## Supplementary information

Supplementary Information
