# Supplementary material for: Structure-efficiency relationship of photoinduced electron transfer-triggered nitric oxide releasers
Source: Sci Rep. 2019 Feb 5;9:1430. doi: 10.1038/s41598-018-38252-5 (PMC6363743; doi:10.1038/s41598-018-38252-5)

Supplementary Information

Structure-activity relationship of photoinduced electron transfer-triggered nitric oxide releasers

Naoya Ieda, Yumina Oka, Toshitada Yoshihara, Seiji Tobita, Takahiro Sasamori, Mitsuyasu Kawaguchi, and Hidehiko Nakagawa

Contents

1. General information
2. Experimental section
    A. Synthesis
    B. ^{1}H NMR charts of NO-Rosa2–6, and NO-Rosa-Gal
    C. Absorption spectra
    D. Fluorescence spectra
    E. Fluorescence quantum yield
    F. Fluorescence lifetime
    G. NO measurement using an NO electrode
    H. NO release quantum yield
    I. Electrochemical analysis
    J. Photodecomposition detection using LC-MS
    K. Tracking enzymatic hydrolysis using HPLC
    L. Light and enzyme specific NO release in cellular condition
3. Figures, Table, and Scheme for supporting data

1. General information

Proton nuclear magnetic resonance spectra (^1H NMR) and carbon nuclear magnetic resonance spectra (^{13}C NMR) were recorded on a JEOL JNM-LA500, JEOL JNM-A500, Varian VNMRS 500, or Bruker AVANCE600 spectrometer in the indicated solvent. Chemical shifts (δ) are reported in parts per million relative to the internal standard, tetramethylsilane. Elemental analysis was performed with Yanaco CHN CORDER NT-5 analyzer, and all values were within ±0.4 % of the calculated values. Fast atom bombardment (FAB) mass spectra were recorded on a JEOL JMS-SX102A mass spectrometer. Ultraviolet-visible-light absorption spectra were recorded on an Agilent 8453 spectrometer. Fluorescence spectra were recorded on RF-5300 PC (Shimadzu). Irradiation was conducted with Asahi Spectra irradiating apparatus (MAX-303). All other reagents and solvents were purchased from Aldrich, Tokyo Kasei Kogyo, FUJIFILM Wako Pure Chemical Corp., Nacalai Tesque, Kanto Kagaku, Kishida Kagaku, Junsei Kagaku or Dojindo, and used without purification. β-D-Galactosidase was purchased from FUJIFILM Wako Chemical Corp. (cat# 072-04141). Flash column chromatography was performed using silica gel 60 supplied by Taiko Shoji. MPLC purification was performed using YFLC-Wprep2XY-S (Yamazen).

2. Experimental section

A. Synthesis

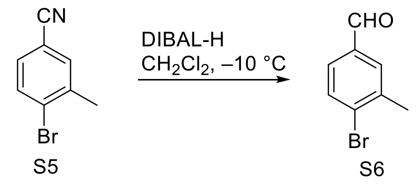

To a solution of S5 (1.96 g, 10.0 mmol) in dry CH_2Cl_2 (20 mL) was added dropwise (1.0 mL/min, via a syringe pump) a solution of 1.0 M DIBAL-H in *n*-hexane (10.5 mL, 10.5 mmol, 1.1 equiv.) at −10 °C under a N_2 balloon. The mixture was stirred for 25 min, and then the mixture was quenched with 2 N HCl (50 mL) and sat. Rochelle's salt aqueous solution (50 mL). The resulting precipitate was removed by filtration and the filtrate was extracted with CH_2Cl_2 three times. The organic layer was washed with brine and dried over Na_2SO_4. Filtration, evaporation *in vacuo*, and purification of the residue by silica gel flash chromatography (*n*-hexane/AcOEt = 20/1 → 10/1) gave 1.65 g (83%) of S6 as a clear oil: ^{1}H NMR ($CDCl_3$, 500 MHz, δ; ppm) 9.96 (1H, s), 7.73 (1H, d, J = 1.3 Hz), 7.71 (1H, d, J = 8.4 Hz), 7.55 (1H, dd, J = 1.3 Hz, 8.4 Hz), 2.48 (3H, s).

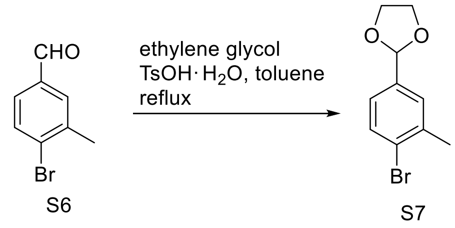

To a solution of S6 (1.65 g, 8.31 mmol) in toluene (20 mL) were added ethylene glycol (4.65 mL, 83.2 mmol, 10 equiv.) and TsOH·H_2O (159 mg, 0.836 mmol, 0.11 equiv.). The reaction mixture was stirred at reflux temperature for 11.5 hr, then cooled, diluted with AcOEt, and washed with sat. $NaHCO_3$ (20 mL×3). The aqueous layer was extracted with AcOEt. The combined organic layer was washed with brine and dried over Na_2SO_4. Filtration, evaporation *in vacuo*, and purification of the residue by silica gel flash chromatography (*n*-hexane/AcOEt = 20/1 → 15/1 → 10/1 → 9/1) gave 1.49 g (74%) of S7 as a colorless oil: ^{1}H NMR ($CDCl_3$, 500 MHz, δ; ppm) 7.53 (1H, d, J = 8.2 Hz), 7.35 (1H, d, J = 1.8 Hz), 7.16 (1H, dd, J = 1.8 Hz, 8.2 Hz), 5.75 (1H, s), 4.15–4.00 (4H, m), 2.41 (3H, s).

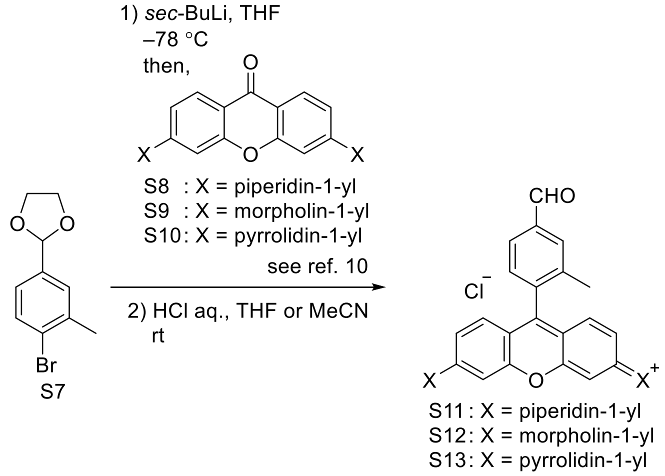

Preparation of S11: A solution of S7 (1.49 g, 6.11 mmol, 3.3 equiv.) in dry THF (30 mL) was stirred at –78 °C under an argon balloon. To the solution was added dropwise a 1.04 M solution of *sec*-BuLi in *n*-hexane/cyclohexane (5.6 mL, 5.82 mmol, 3.1 equiv.), and stirring was continued for 1 hr. Then, a solution of S8 (673 mg, 1.86 mmol) in dry THF (10 mL×2) was added dropwise. The reaction mixture was stirred at room temperature for a further 2 hr, then the reaction was quenched with AcOH (1 mL), and the mixture was evaporated *in vacuo*. The residue was dissolved in THF (20 mL), and 2 N HCl (20 mL) was added. The reaction mixture was stirred at room temperature for 12 hr, then diluted with water (100 mL) and extracted with CH_2Cl_2 three times. The organic layer was dried over Na_2SO_4. Filtration, evaporation *in vacuo*, and purification of the residue by silica gel flash chromatography (CH_2Cl_2/MeOH = 20/1 → 15/1 → 10/1 → 5/1) gave 837 mg (90%) of S11 as a purple solid: ^{1}H NMR (DMSO-d_6, 500 MHz, δ; ppm) 8.07 (1H, s), 8.00 (1H, d, J = 7.7 Hz), 7.56 (1H, d, J = 7.8 Hz), 7.30 (2H, dd, J = 2.2 Hz, 9.6 Hz), 7.19 (2H, d, J = 2.2 Hz), 7.00 (2H, d, J = 9.6 Hz), 3.77 (8H, brm), 2.12 (3H, s), 1.71–1.65 (12H, brm).

Preparation of S12: A solution of S7 (376 mg, 1.55 mmol, 3.3 equiv.) in dry THF (6 mL) was stirred at –78 °C under an argon balloon for an hour, and 1.01 M *sec*-BuLi in *n*-hexane/cyclohexane (1.4 mL, 1.41 mmol, 3.0 equiv.) was added dropwise. Stirring was continued at –78 °C under an argon balloon for an hour, and then a slurry of S9 (172 mg, 0.469 mmol) in dry THF (4 mL×2) was added. The reaction mixture was removed from the low-temperature bath and stirred at room temperature for 25 min. The reaction was confirmed to be complete by ESI-MS. AcOH (0.2 mL) was added, and the mixture was concentrated *in vacuo*. The residue was dissolved in MeCN (5 mL) and 2 N HCl (5 mL), and the solution was stirred at room temperature for 15 min. The reaction was confirmed to be complete by ESI-MS, then the mixture was diluted with water (40 mL) and extracted with CH_2Cl_2 three times. The organic layer was dried over Na_2SO_4. Filtration, evaporation *in vacuo*, and purification of the residue by silica gel flash chromatography (CH_2Cl_2/MeOH = 15/1 → 12/1 → 9/1

→ 8/1) gave 226 mg (95%) of S12 as a purple solid: ^{1}H NMR (DMSO-d_6, 500 MHz, δ; ppm) 10.16 (1H, s), 8.08 (1H, s), 8.01 (1H, d, J = 8.0 Hz), 7.57 (1H, d, J = 8.0 Hz), 7.33 (2H, dd, J = 2.4 Hz, 9.7 Hz), 7.26 (2H, d, J = 2.4 Hz), 7.08 (2H, d, J = 9.7 Hz), 3.77 (16H, brs), 2.11 (3H, s).

Preparation of S13: A solution of S7 (642 mg, 2.64 mmol, 3.3 equiv.) in dry THF (9 mL) was stirred at –78 °C under an argon balloon for an hour, then 1.05 M *sec*-BuLi in *n*-hexane/cyclohexane (2.4 mL, 2.52 mmol, 3.1 equiv.) was added dropwise. The reaction mixture was stirred at –78 °C under an argon balloon for an hour, and then a slurry of S10 (267 mg, 0.798 mmol) in dry THF (4.5 mL×2) was added. The reaction mixture was removed from the low-temperature bath and stirred at room temperature for 40 min. The reaction was confirmed to be complete by ESI-MS, and 2 N HCl (18 mL) was added. The mixture was stirred at room temperature for 20 min, then diluted with water (60 mL) and brine (30 mL), and extracted with CH_2Cl_2 three times. The organic layer was dried over Na_2SO_4. Filtration, evaporation *in vacuo*, and purification of the residue on a Yamazen MPLC system (CH_2Cl_2/MeOH = 94/6 → 90/10 → 80/20) gave 359 mg (95%) of S13 as a purple solid: ^{1}H NMR (DMSO-d_6, 500 MHz, δ; ppm) 10.16 (1H, s), 8.07 (1H, s), 8.01 (1H, d, J = 7.8 Hz), 7.58 (1H, d, J = 7.8 Hz), 7.03 (2H, d, 9.2 Hz), 6.98 (2H, dd, J = 2.2 Hz, 9.2 Hz), 6.87 (2H, d, J = 2.2 Hz), 3.60 (8H, brs), 2.10 (3H, s), 2.05 (8H, brs).

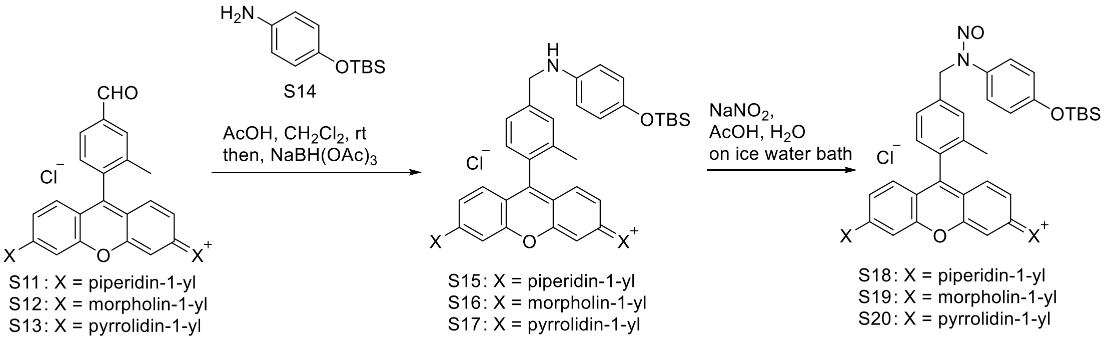

S11 : X = piperidin-1-yl
S12: X = morpholin-1-yl
S13: X = pyrrolidin-1-yl

S15: X = piperidin-1-yl
S16: X = morpholin-1-yl
S17: X = pyrrolidin-1-yl

S18: X = piperidin-1-yl
S19: X = morpholin-1-yl
S20: X = pyrrolidin-1-yl

Preparation of S15: To a solution of S14 (143 mg, 0.640 mmol, 1.1 equiv.) in CH_2Cl_2 (4 mL) was added S11 (292 mg, 0.583 mmol), followed by AcOH (0.2 mL). The mixture was stirred at room temperature for 30 min, and NaBH(OAc)$_3$ (371 mg, 1.75 mmol, 3.0 equiv.) was added. Stirring was continued for an hour, then the mixture was quenched with 0.1 N HCl (20 mL), and extracted with CH_2Cl_2 three times. The organic layer was dried over Na_2SO_4. Filtration, evaporation *in vacuo* and purification of the residue by silica gel flash chromatography (CH_2Cl_2/MeOH = 10/1 → 8/1) gave 243 mg (59%) of S15 as a purple solid: ^{1}H NMR (DMSO-d_6, 500 MHz, δ; ppm) 7.51 (1H, s), 7.44 (1H, d, J = 7.9 Hz), 7.31 (2H, dd, J = 2.3 Hz, 9.6 Hz), 7.25 (1H, d, J = 7.6 Hz), 7.17 (2H, d, J = 2.3 Hz), 7.02 (2H, d, J = 9.5 Hz), 6.60 (4H, dd, J = 2.3 Hz, 9.6 Hz), 5.95 (1H, t, J = 6.0 Hz), 4.32 (2H, d, J = 6.0 Hz), 3.76 (8H, brm), 2.01 (3H, s), 1.70–1.64 (12H, brm), 0.93 (9H, s), 0.13 (6H, s).

Preparation of S16: To a solution of S12 (218 mg, 0.432 mmol) in CH_2Cl_2 (10 mL) was added S14 (111 µL, 0.474 mmol, 1.1 equiv.) and AcOH (1 mL). The mixture was stirred at room temperature for an hour, and $NaBH(OAc)_3$ (261 mg, 1.23 mmol, 3.0 equiv.) was added. Stirring was continued for 15 min, then the mixture was quenched with 0.1 N HCl (40 mL) and extracted with CH_2Cl_2 three times. The organic layer was dried over Na_2SO_4. Filtration, evaporation *in vacuo*, and purification of the residue by silica gel flash chromatography (CH_2Cl_2/MeOH = 15/1 → 12/1 → 10/1 → 9/1 → 9/2) gave 172 mg (56%) of S16 as a purple solid: ^{1}H NMR (DMSO-d_6, 500 MHz, δ; ppm) 7.52 (1H, s), 7.46 (1H, d, J = 7.8 Hz), 7.35 (2H, dd, J = 2.4 Hz, 9.6 Hz), 7.26 (1H, d, J = 7.8 Hz), 7.24 (2H, d, J = 2.4 Hz), 7.10 (2H, d, J = 9.6 Hz), 6.63 (2H, d, J = 8.8 Hz), 6.57 (2H, d, J = 8.8 Hz), 5.94 (1H, t, J = 6.0 Hz), 4.32 (1H, d, J = 6.0 Hz), 3.77 (16 H, brs), 2.00 (3H, s), 0.93 (9H, s), 0.13 (6H, s).

Preparation of S18: To a solution of S15 (200 mg, 0.283 mmol) in AcOH (3 mL) was added a solution of $NaNO_2$ (21 mg, 0.304 mmol, 1.1 equiv.) in water (3 mL) on an ice-water bath. The mixture was stirred on the ice-water bath for 15 min, then diluted with water (30 mL) and brine (70 mL), and extracted with CH_2Cl_2 three times. The organic layer was dried over Na_2SO_4. Filtration, evaporation *in vacuo*, and purification of the residue by silica gel flash chromatography (CH_2Cl_2/MeOH = 15/1 → 10/1 → 8/1) gave 186 mg (89%) of S18 as a purple solid: ^{1}H NMR (DMSO-d_6, 500 MHz, δ; ppm) 7.60 (2H, d, J = 8.9 Hz), 7.28 (2H, dd, J = 2.3 Hz, 9.6 Hz), 7.24 (1H, s), 7.23 (1H, d, J = 8.0 Hz), 7.16 (2H, d, J = 2.3 Hz), 7.12 (1H, d, J = 8.0 Hz), 7.03 (2H, d, J = 8.9 Hz), 6.95 (2H, d, J = 9.6 Hz), 5.41 (2H, s), 3.75 (8H, brm), 1.97 (3H, s), 1.70–1.64 (12H, m), 0.97 (9H, s), 0.23 (6H, s).

Preparation of S19: To a solution of S16 (172 mg, 0.241 mmol) in AcOH (5 mL) was added a solution of $NaNO_2$ (18 mg, 0.261 mmol, 1.1 equiv.) in water (5 mL) on an ice-water bath. The mixture was stirred on the ice-water bath for 15 min, then diluted with water (20 mL) and brine (20 mL), and extracted with CH_2Cl_2 three times. The organic layer was dried over Na_2SO_4. Filtration, evaporation *in vacuo*, and purification of the residue by silica gel flash chromatography (CH_2Cl_2/MeOH = 15/1 → 12/1 → 10/1 → 9/1 → 9/2 → 4/1) gave 158 mg (88%) of S19 as a purple solid: ^{1}H NMR (DMSO-d_6, 500 MHz, δ; ppm) 7.60 (2H, d, J = 9.0 Hz), 7.32 (2H, dd, J = 2.4 Hz, 9.7 Hz), 7.25–7.23 (4H, m), 7.13 (1H, d, J = 8.6 Hz), 7.05–7.03 (4H, m), 5.41 (2H, s), 3.76 (16H, brs), 1.96 (3H, s), 0.97 (9H, s), 0.23 (6H, s).

Preparation of S20: To a solution of S13 (142 mg, 0.300 mmol) in CH_2Cl_2 (6 mL) was added S14 (77 µL, 0.330 mmol, 1.1 equiv.) and AcOH (0.6 mL). The mixture was stirred for an hour, then $NaBH(OAc)_3$ (191 mg, 0.901 mmol, 3.0 equiv.) was added. Stirring was continued for 30 min, then the mixture was quenched with 0.1 N HCl (30 mL) and extracted with CH_2Cl_2 three times. The organic

layer was dried over Na_2SO_4, filtered, and concentrated *in vacuo* to obtain crude S17, which was dissolved in AcOH (3 mL). To this solution was added a solution of $NaNO_2$ (25 mg, 0.362 mmol, 1.2 equiv.) in water (3 mL) on an ice-water bath. The mixture was stirred on the ice-water bath for 15 min, then quenched with 0.1 N HCl (30 mL) and brine (30 mL), and extracted with CH_2Cl_2 three times. The organic layer was dried over Na_2SO_4. Filtration, evaporation *in vacuo*, and purification of the residue on a Yamazen MPLC system (CH_2Cl_2/MeOH = 94/6 → 90/10 → 80/20) gave 163 mg (77%) of S20 as a purple solid: ^{1}H NMR (DMSO-d_6, 500 MHz, δ; ppm) 7.60 (1H, d, J = 9.1 Hz), 7.49 (1H, d, J = 9.0 Hz), 7.25–7.22 (2H, m), 7.13 (1H, d, J = 7.6 Hz), 7.03 (1H, d, J = 9.0 Hz), 6.97 (4H, brs), 6.93 (1H, d, J = 9.1 Hz), 6.83 (2H, brs), 5.41 (1H, s), 5.38 (1H, s), 3.58 (8H, brs), 2.04 (8H, brs), 1.95 (3H, s), 0.97–0.84 (9H, m), 0.23 (4H, s), –0.04 (2H, s).

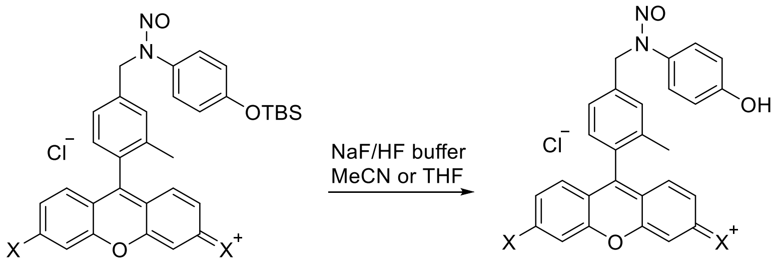

S18: X = piperidin-1-yl
S19: X = morpholin-1-yl
S20: X = pyrrolidin-1-yl

NO-Rosa2: X = piperidin-1-yl
NO-Rosa3: X = morpholin-1-yl
NO-Rosa4: X = pyrrolidin-1-yl

Preparation of NO-Rosa2: To a solution of S18 (173 mg, 0.235 mmol) in THF (5 mL) was added 0.1 M NaF/HF buffer (3 mL). The mixture was stirred at room temperature for 2 hr, then water (40 mL) was added, and the mixture was extracted with CH_2Cl_2 three times. The organic layer was dried over Na_2SO_4. Filtration, evaporation *in vacuo*, and purification of the residue by silica gel flash chromatography (CH_2Cl_2/MeOH = 15/1 → 12/1 → 10/1 → 8/1 → 7/1) gave 116 mg (79%) of NO-Rosa2 as a purple solid: ^{1}H NMR (DMSO-d_6, 500 MHz, δ; ppm) 9.89 (1H, s), 7.50 (2H, d, J = 8.7 Hz), 7.29 (2H, dd, J = 2.4 Hz, 9.6 Hz), 7.23–7.21 (2H, m), 7.15 (2H, d, J = 2.4 Hz), 7.11 (1H, d, J = 8.3 Hz), 6.95–6.92 (4H, m), 5.38 (2H, s), 3.76–3.74 (8H, m), 1.96 (3H, s), 1.71–1.64 (12H, m); ^{13}C NMR (DMSO-d_6, 150 MHz, δ; ppm) 157.63, 157.15, 156.03, 155.39, 136.49, 135.86, 132.88, 130.94, 130.42, 129.25, 129.06, 124.66, 122.36, 115.87, 115.11, 113.00, 96.77, 69.68, 48.34, 47.15, 25.60, 23.61, 19.04; MS (ESI) m/z: 587 (M^+), 557 ($[M–NO]^+$); Anal. Calcd. for $C_{37}H_{39}ClN_4O_3 \cdot 5/2H_2O$: C, 66.50; H, 6.64; N, 8.38. Found: C, 66.50; H, 6.41; N, 8.63.

Preparation of NO-Rosa3: To a solution of S19 (158 mg, 0.213 mmol) in THF (6 mL) and MeCN (3 mL) was added 0.1 M NaF/HF buffer (3 mL). The mixture was stirred at room temperature for 2.5 hr, then water (20 mL) and brine (20 mL) were added, and the whole was extracted with CH_2Cl_2 three times. The organic layer was dried over Na_2SO_4. Filtration, evaporation *in vacuo*, and purification of

the residue by silica gel flash chromatography (CH_2Cl_2/MeOH = 15/1 → 12/1 → 10/1 → 4/1) gave 121 mg (91%) of NO-Rosa3 as a purple solid: ^{1}H NMR (DMSO-d_6, 500 MHz, δ; ppm) 9.89 (1H, s), 7.50 (2H, d, J = 9.1 Hz), 7.35 (2H, dd, J = 2.3 Hz, 9.7 Hz), 7.24–7.22 (4H, m), 7.13 (1H, d, J = 8.0 Hz), 7.03 (2H, d, J = 9.6 Hz), 6.92 (2H, d, J = 9.1 Hz), 5.38 (2H, s), 3.76 (16H, brs), 1.95 (3H, s); ^{13}C NMR (DMSO-d_6, 150 MHz, δ; ppm) 157.64, 157.13, 156.80, 156.74, 136.64, 135.86, 132.90, 131.00, 130.26, 129.24, 129.09, 124.65, 122.36, 115.87, 115.21, 113.72, 97.13, 65.69, 54.79, 47.18, 46.99, 19.02; MS (ESI) m/z: 591 (M^+), 561 ($[M–NO]^+$); Anal. Calcd. for $C_{35}H_{35}ClN_4O_5 \cdot 5/2H_2O$: C, 62.54; H, 6.00; N, 8.34. Found: C, 62.52; H, 5.93; N, 8.37.

Preparation of NO-Rosa4: To a solution of S20 (163 mg, 0.230 mmol) in MeCN (4 mL) was added 0.1 M NaF/HF buffer (4 mL). The mixture was stirred at room temperature for 2 hr, then brine (40 mL) was added, and the whole was extracted with CH_2Cl_2 three times. The organic layer was dried over Na_2SO_4. Filtration, evaporation *in vacuo*, and purification of the residue on a Yamazen MPLC system (CH_2Cl_2/MeOH = 94/6 → 90/10 → 80/20) gave 163 mg (77%) of NO-Rosa4 as a purple solid: ^{1}H NMR (DMSO-d_6, 500 MHz, δ; ppm) 9.93 (1H, brs), 7.49 (2H, d, J = 8.7 Hz), 7.24–7.22 (2H, m), 7.13 (1H, d, J =7.8 Hz), 6.98–6.97 (4H, m), 6.93 (2H, d, J = 8.7 Hz), 6.83 (2H, d, J = 1.5 Hz), 5.38 (2H, s), 3.58 (8H, brs), 2.04 (8H, brs), 1.95 (3H, s); ^{13}C NMR (DMSO-d_6, 150 MHz, δ; ppm) 157.17, 156.74, 156.13, 154.17, 136.44, 135.79, 132.88, 130.67, 130.59, 129.20, 129.05, 127.37, 124.67, 122.36, 115.88, 115.66, 112.69, 96.62, 48.78, 47.16, 24.70, 24.54, 18.95; MS (ESI) m/z: 559 (M^+), 529 ($[M–NO]^+$); Anal. Calcd. for $C_{35}H_{35}ClN_4O_3 \cdot 5/2H_2O$: C, 65.67; H, 6.30; N, 8.75. Found: C, 65.30; H, 6.12; N, 8.62.

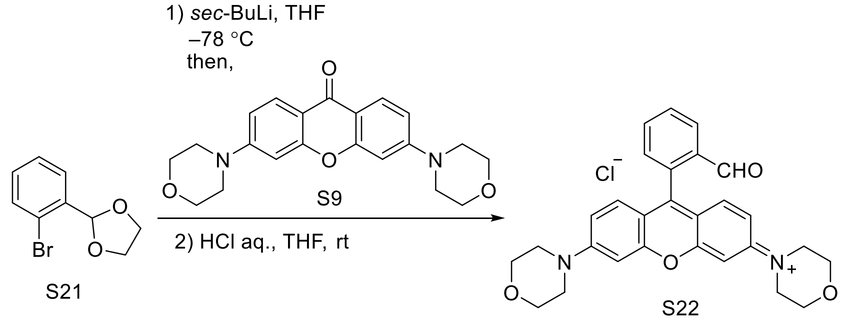

A solution of S21 (494 µL, 3.30 mmol, 3.3 equiv.) in dry THF (12 mL) was stirred at –78 °C under an argon balloon for an hour, then 1.01 M *sec*-BuLi in *n*-hexane/cyclohexane (3.0 mL, 3.03 mmol, 3.0 equiv.) was added dropwise. Stirring was continued at –78 °C under an argon balloon for an hour, and then a solution of S9 (366 mg, 0.999 mmol) in dry THF (8 mL×2) was added. The reaction mixture was removed from the low-temperature bath and stirred at room temperature for 30 min. The reaction was confirmed to be complete by ESI-MS, then the mixture was quenched with AcOH (1 mL), and evaporated *in vacuo*. The residue was dissolved in MeCN (10 mL) and 2 N HCl (10 mL). This solution

was stirred for 40 min, then diluted with water (50 mL), and extracted with CH_2Cl_2 three times. The organic layer was dried over Na_2SO_4. Filtration, evaporation *in vacuo*, and purification of the residue by silica gel flash chromatography (CH_2Cl_2/MeOH = 16/1 → 10/1 → 5/1) gave a mixture of aldehyde and methyl hemiacetal. To hydrolyze the hemiacetal, the mixture was dissolved in MeCN (10 mL) and 2 N HCl (10 mL), stirred for a few minutes, diluted with water (50 mL), and extracted with CH_2Cl_2 three times. The organic layer was dried over Na_2SO_4. Filtration, and evaporation of the residue *in vacuo* gave 429 mg (87%) of S22 as a purple solid: ^{1}H NMR (DMSO-d_6, 500 MHz, δ; ppm) 9.86 (1H, s), 8.26 (1H, dd, J = 1.3 Hz, 7.3 Hz), 7.99–7.92 (2H, m), 7.56 (1H, dd, J = 1.3 Hz, 7.3 Hz), 7.25 (2H, dd, J = 2.4 Hz, 9.6 Hz), 7.18 (2H, d, J = 2.4 Hz), 6.97 (2H, d, J = 9.6 Hz), 3.76 (8H, t, J = 5.2 Hz), 1.73–1.64 (12H, m).

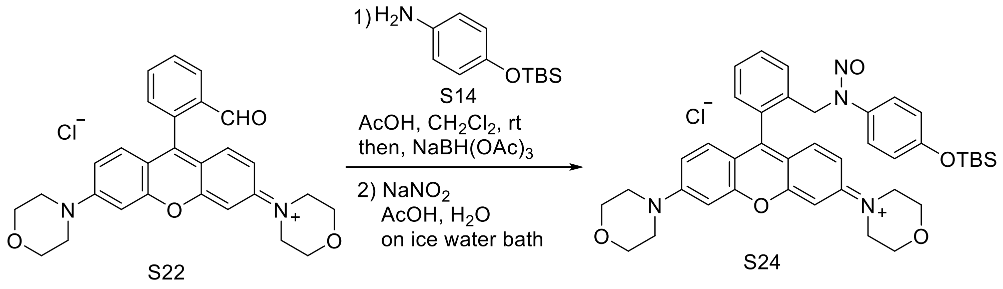

To a solution of S22 (362 mg, 0.737 mmol) in CH_2Cl_2 (15 mL) was added S14 (190 µL, 0.811 mmol, 1.1 equiv.) and AcOH (1.5 mL). The mixture was stirred for an hour, and $NaBH(OAc)_3$ (462 mg, 2.18 mmol, 3.0 equiv.) was added. Stirring was continued for 15 min, then the mixture was quenched with 0.1 N HCl (50 mL) and extracted with CH_2Cl_2 three times. The organic layer was dried over Na_2SO_4, filtered, and concentrated *in vacuo*. The residue was dissolved into AcOH (8 mL). To this solution was added a solution of $NaNO_2$ (56 mg, 0.812 mmol, 1.1 equiv.) in water (8 mL) on an ice-water bath. The mixture was stirred on an ice-water bath for 15 min, then quenched with 0.1 N HCl (80 mL) and extracted with CH_2Cl_2 three times. The organic layer was dried over Na_2SO_4. Filtration, evaporation *in vacuo*, and purification of the residue by silica gel flash chromatography (CH_2Cl_2/MeOH = 15/1 → 10/1 → 4/1) gave 354 mg (66%) of a mixture of S24 as a purple solid: ^{1}H NMR (DMSO-d_6, 500 MHz, δ; ppm) 7.62–7.54 (2H, m), 7.36–7.32 (4H, m), 7.21 (2H, d, J = 2.6 Hz), 7.15 (2H, d, J = 9.1 Hz), 7.03 (2H, d, J = 9.3 Hz), 6.82 (2H, d, J = 9.1 Hz), 4.96 (2H, s), 3.79 (16H, brs), 0.95 (9H, s), 0.20 (6H, s).

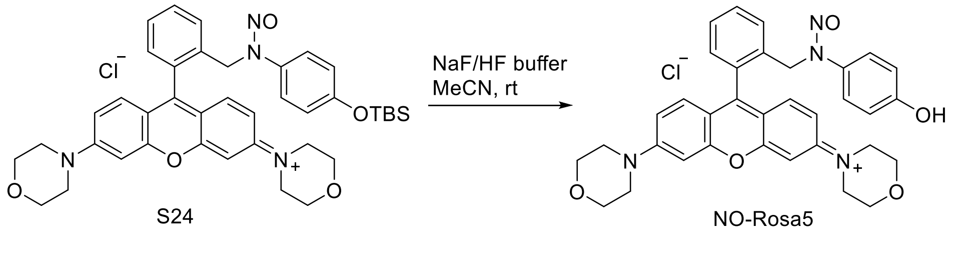

To a solution of S24 (354 mg, 0.487 mmol) in MeCN (7 mL) was added 0.1 M NaF/HF buffer (7 mL). The mixture was stirred at room temperature for 2.5 hr, then water (35 mL) and brine (35 mL) were added, and the whole was extracted with CH_2Cl_2 three times. The organic layer was dried over Na_2SO_4. Filtration, evaporation *in vacuo*, and purification of the residue by silica gel flash chromatography (CH_2Cl_2/MeOH = 15/1 → 9/1 → 4/1) gave 216 mg (72%) of NO-Rosa5 as a purple solid: ^{1}H NMR (DMSO-d_6, 500 MHz, δ; ppm) 9.85 (1H, s), 7.62–7.53 (2H, m), 7.21 (2H, d, J = 2.3 Hz), 7.02 (2H, d, J = 8.8 Hz), 6.99 (2H, d, J = 9.5 Hz), 6.70 (2H, d, J = 9.0 Hz), 4.95 (2H, s), 3.79 (16H, s); ^{13}C NMR (DMSO-d_6, 150 MHz, δ; ppm) 157.58, 157.07, 156.82, 155.12, 133.05, 131.90, 130.90, 130.55, 130.27, 129.44, 127.88, 122.00, 115.54, 115.12, 113.95, 97.01, 65.72, 54.81, 47.00, 44.88; MS (ESI) m/z: 577 (M^+), 547 ($[M–NO]^+$); Anal. Calcd. for $C_{34}H_{33}ClN_4O_5 \cdot 5/2H_2O$: C, 62.05; H, 5.82; N, 8.51. Found: C, 62.26; H, 5.73; N, 8.52.

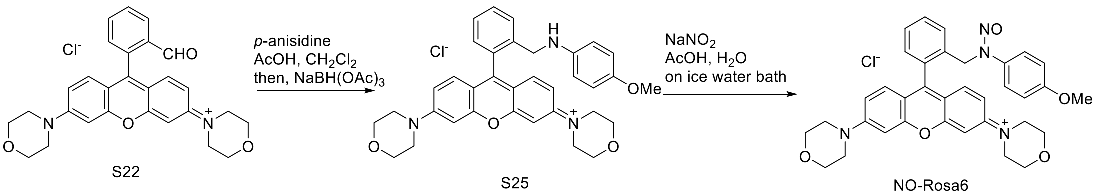

To a solution of S22 (99 mg, 0.202 mmol) and *p*-anisidine (28 mg, 0.227 mmol, 1.1 equiv.) in CH_2Cl_2 (4 mL) was added AcOH (0.4 mL). The mixture was stirred for an hour, then $NaBH(OAc)_3$ (132 mg, 0.623 mmol, 3.1 equiv.) was added. Stirring was continued for 40 min, then the mixture was quenched with 0.1 N HCl (20 mL), and extracted with CH_2Cl_2 three times. The organic layer was dried over Na_2SO_4. Filtration, and evaporation *in vacuo* gave crude S25, which was dissolved in AcOH (2 mL). To this solution was added a solution of $NaNO_2$ (17 mg, 0.246 mmol, 1.2 equiv.) in water (2 mL) on an ice-water bath. The mixture was stirred for 25 min, then 0.1 N HCl (20 mL) and brine (30 mL) were added, and the whole was extracted with CH_2Cl_2 three times. The organic layer was dried over Na_2SO_4. Filtration, evaporation *in vacuo*, and purification of the residue on a Yamazen MPLC system (CH_2Cl_2/MeOH = 94/6 → 90/10 → 80/20) gave 83 mg (66%) of NO-Rosa6 as a purple solid: ^{1}H NMR ($CDCl_3$, 500 MHz, δ; ppm) 7.57–7.47 (2H, m), 7.30–7.24 (7H, m), 7.21–7.18 (3H, m), 6.88 (2H, d, J = 9.1 Hz), 4.86 (2H, s), 3.93 (8H, t, J = 4.8 Hz), 3.84 (3H, s), 3.82 (8H, t, J = 4.8 Hz); ^{13}C NMR ($CDCl_3$, 150 MHz, δ; ppm) 159.23, 158.21, 157.46, 156.25, 134.38, 133.03, 131.61, 130.81, 130.45, 129.49, 128.42, 128.13, 121.39, 115.49, 114.81, 114.73, 98.35, 66.43, 55.80, 47.67, 46.58; MS (FAB) m/z: 591 (M^+), 561 ($[M–NO]^+$); Anal. Calcd. for $C_{35}H_{35}ClN_4O_5 \cdot 5/2H_2O$: C, 62.54; H, 6.00; N, 8.34. Found: C, 62.74; H, 6.00; N, 8.34.

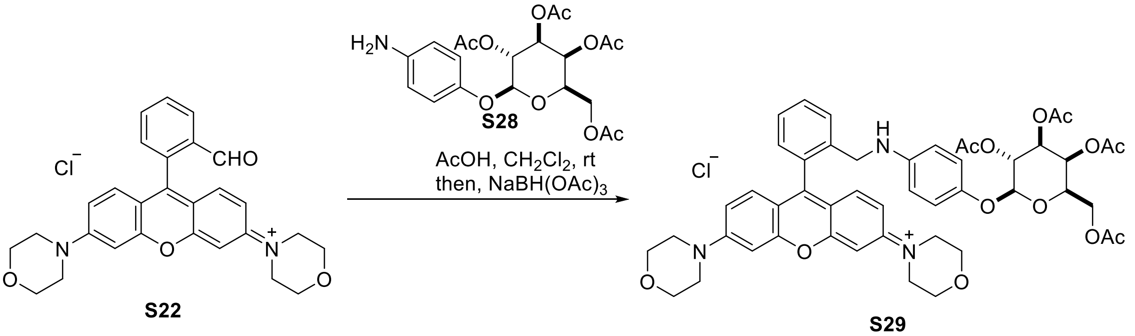

S22 **S29**

To a solution of S22 (70.5 mg, 0.144 mmol) and S28 (76.5 mg, 0.174 mmol, 1.2 equiv.) in CH_2Cl_2 (6 mL) was added AcOH (0.5 mL). The mixture was stirred for an hour at room temperature, then $NaBH(OAc)_3$ (93.5 mg, 0.441 mmol, 3.1 equiv.) was added. Stirring was continued for 22.5 hr at room temperature. The reaction was confirmed to be complete by ESI-MS, then the mixture was quenched with 2 N HCl and brine, and extracted with CH_2Cl_2 three times. The organic layer was dried over Na_2SO_4. Filtration, evaporation *in vacuo*, and purification of the residue on a Yamazen MPLC system (CH_2Cl_2/MeOH = 94/6 → 90/10 → 80/20) gave 129 mg (98%) of S29 as a purple solid: ^{1}H NMR (DMSO-d_6, 500 MHz, δ; ppm) 7.48 (1H, d, J = 9.0 Hz), 7.25 (1H, t, J = 7.5 Hz), 7.13 (1H, t, J = 7.8 Hz), 6.69–6.65 (7H, m), 6.55–6.48 (2H, m), 6.43 (2H, d, J = 10 Hz), 5.25 (1H, d, J = 3.5 Hz), 5.13–5.05 (5H, m), 4.25 (1H, t, J = 6.8 Hz), 4.10–4.06 (1H, m), 4.01–3.97 (1H, m), 3.69 (8H, s), 3.09 (8H, s), 2.11 (3H, s), 2.00 (3H, s), 1.91 (3H, s), 1.86 (3H, s).

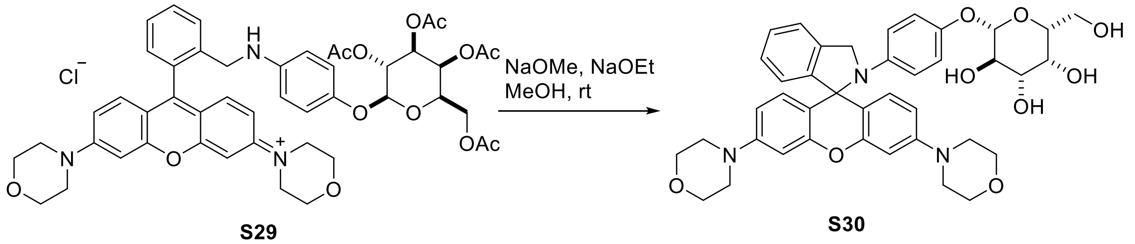

S29 **S30**

A solution of S29 (103 mg, 0.113 mmol) and NaOMe (3.2 mg, 0.0592 mmol, 0.52 equiv.) in dry MeOH (10 mL) was stirred at room temperature under a N_2 balloon. The mixture was stirred for 24 hr, then 20% NaOEt in EtOH (10 μL, 0.0232 mmol, 0.21 equiv.) was added. Stirring was continued for 3 hr. The reaction was confirmed to be complete by ESI-MS, then the mixture was quenched with brine, and extracted with CH_2Cl_2 three times. The organic layer was dried over Na_2SO_4. Filtration, evaporation *in vacuo* and purification of the residue on a Yamazen MPLC system (CH_2Cl_2/MeOH = 94/6 → 90/10 → 80/20 → 70/30 → 0/100) gave 73 mg (87%) of S30 as a pale purple solid: ^{1}H NMR ($CDCl_3$, 500 MHz, δ; ppm) 7.40 (1H, d, J = 7.1 Hz), 7.21 (1H, t, J = 7.8 Hz), 7.12 (1H, t, J = 8.0 Hz), 6.80 (1H, d, J = 7.7 Hz), 6.77–6.72 (4H, m), 6.66 (2H, s), 6.49 (2H, d, J = 9.0 Hz), 6.46–6.43 (2H, m),

5.05 (2H, s), 4.64 (1H, d, *J* = 7.9 Hz), 4.02 (1H, br), 3.98-3.94 (1H, m), 3.87–3.82 (10H, m), 3.64 (1H, s), 3.57 (1H, s), 3.14 (8H, s).

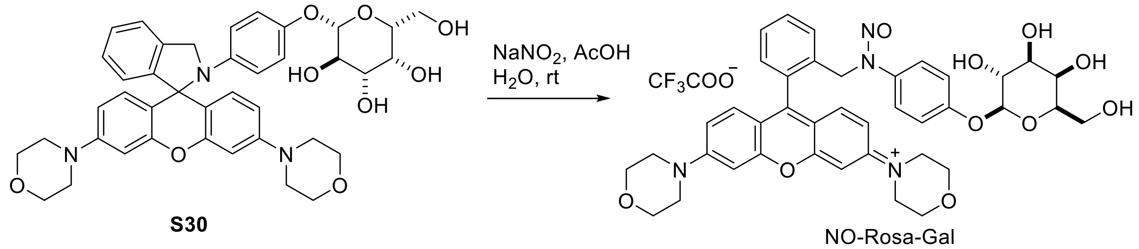

S30 NO-Rosa-Gal

S30 (73.3 mg, 0.0982 mmol) was dissolved in AcOH (5 mL), and a solution of $NaNO_2$ (9.8 mg, 0.142 mmol, 1.4 eq.) in water (5 mL) was added. The mixture was stirred for an hour. The reaction was confirmed to be complete by ESI-MS, then the mixture was evaporated *in vacuo*. The crude product was purified by reverse-phase HPLC (A:B = 80:20 to 20:80 (25 min), A: 0.1% TFA in MilliQ, B; 0.1% TFA in MeCN) to afford 38 mg (49%) of NO-Rosa-Gal as a purple solid: ^{1}H NMR (DMSO-d_6, 500 MHz, δ; ppm) 7.61 (1H, dd, *J* = 6.7 Hz, 6.7 Hz), 7.55 (1H, dd, *J* = 7.4 Hz, 7.4 Hz), 7.42 (1H, d, *J* = 7.8 Hz), 7.32–7.26 (4H, m), 7.18 (2H, s), 7.09–6.99 (3H, m), 6.93 (2H, d, *J* = 9.0 Hz), 6.87 (1H, d, *J* = 9.6 Hz), 5.08 (1H, d, *J* = 16 Hz), 4.90 (1H, d, *J* = 16 Hz), 4.85 (1H, d, *J* = 7.7 Hz), 3.79–3.71 (23H, m), 3.61–3.56 (3H, m); ^{13}C NMR (DMSO-d_6, 150 MHz, δ; ppm) 157.87, 157.61, 157.40, 157.13, 156.67, 156.35, 154.89, 134.01, 132.90, 130.78, 130.63, 130.42, 130.22, 129.36, 129.45, 127.85, 121.41, 116.36, 114.98, 113.76, 100.50, 96.96, 96.83, 75.39, 73.07, 69.98, 67.92, 65.65, 60.26, 46.90, 44.64; Anal. Calcd. for $C_{42}H_{43}F_3N_4O_{12}\cdot 2H_2O$: C, 52.70; H, 4.82; N, 5.59. Found: C, 52.75; H, 4.65; N, 5.38.

B. ^{1}H NMR chart of NO-Rosa2–6, and NO-Rosa-Gal

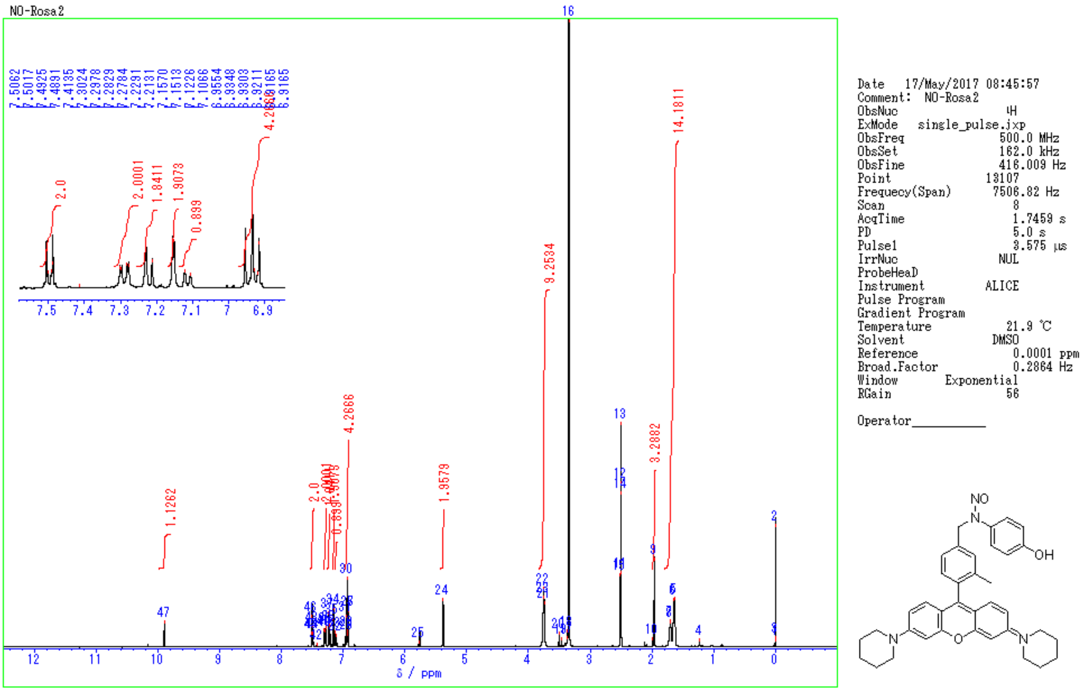

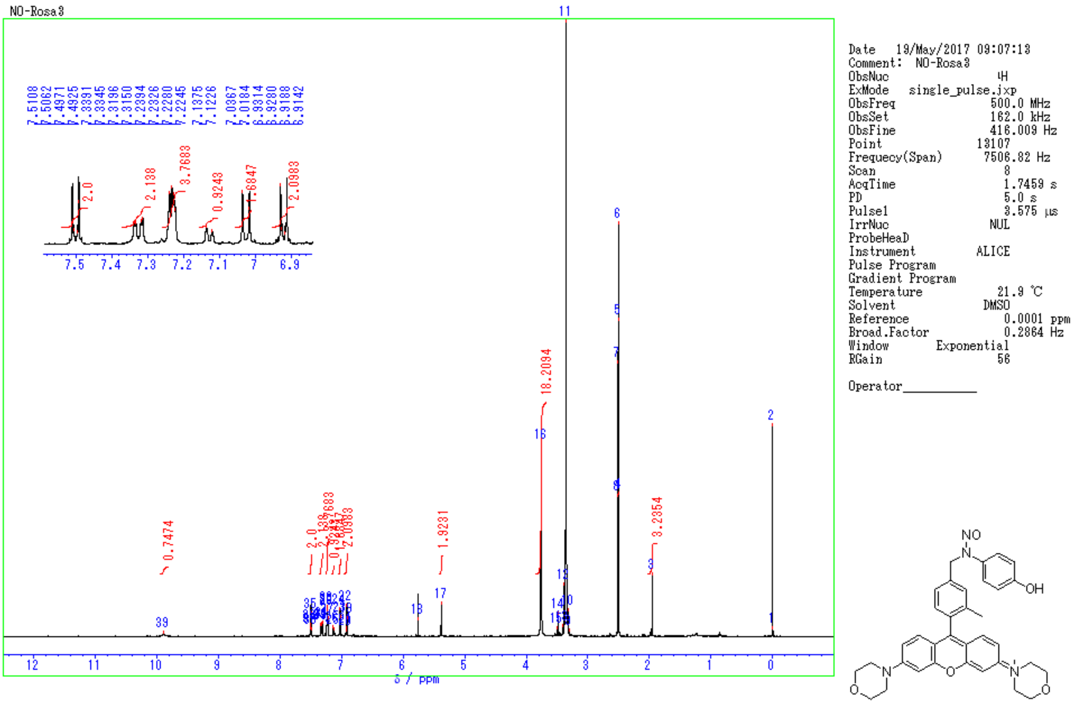

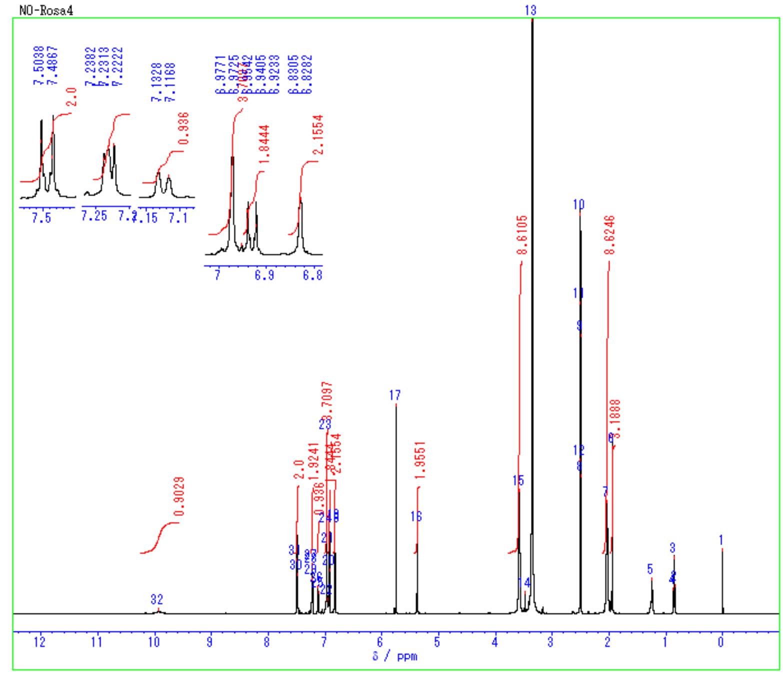

Date 15/Jul/2017 16:13:43
Comment: NO-Rosa4
ObsNuc ¹H
ExMode single_pulse.jxp
ObsFreq 500.0 MHz
ObsSet 162.0 kHz
ObsFine 416.009 Hz
Point 13107
Frequecy(Span) 7506.82 Hz
Scan 8
AcqTime 1.7459 s
PD 5.0 s
Pulse1 3.575 μs
IrrNuc NUL
ProbeHeaD
Instrument ALICE
Pulse Program
Gradient Program
Temperature 23.6 ℃
Solvent DMSO
Reference 0.0 ppm
Broad.Factor 0.2864 Hz
Window Exponential
RGain 56

Operator__________

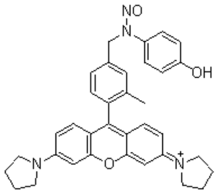

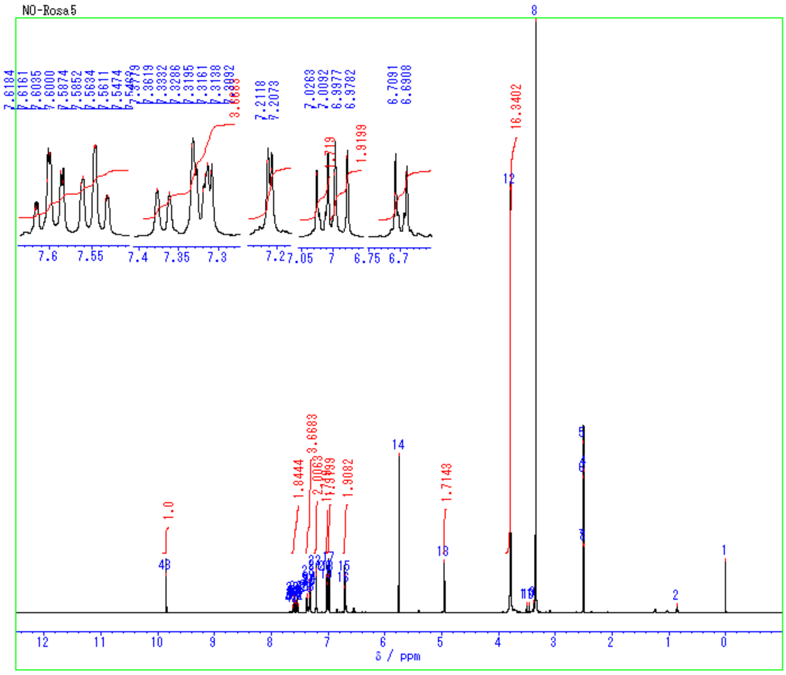

Date 01/Jun/2017 21:00:42
Comment: NO-Rosa5
ObsNuc ¹H
ExMode single_pulse.jxp
ObsFreq 500.0 MHz
ObsSet 162.0 kHz
ObsFine 416.009 Hz
Point 13107
Frequecy(Span) 7506.82 Hz
Scan 8
AcqTime 1.7459 s
PD 5.0 s
Pulse1 3.575 μs
IrrNuc NUL
ProbeHeaD
Instrument ALICE
Pulse Program
Gradient Program
Temperature 22.5 ℃
Solvent DMSO
Reference 0.0 ppm
Broad.Factor 0.2864 Hz
Window Exponential
RGain 56

Operator__________

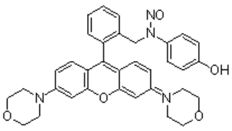

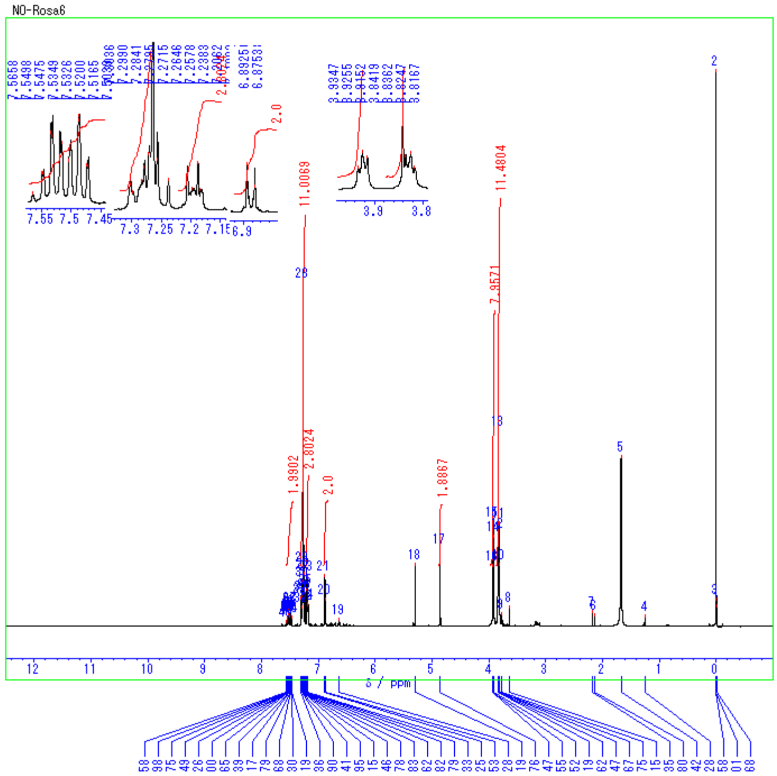

Date 30/Aug/2017 19:53:26
Comment: NO-Rosa6
ObsNuc ¹H
ExMode single_pulse.jxp
ObsFreq 500.0 MHz
ObsSet 162.0 kHz
ObsFine 416.009 Hz
Point 13107
Frequecy(Span) 7506.82 Hz
Scan 8
AcqTime 1.7459 s
PD 5.0 s
Pulse1 3.575 µs
IrrNuc NUL
ProbeHeaD
Instrument ALICE
Pulse Program
Gradient Program
Temperature 22.7 ℃
Solvent $CDCl_3$
Reference 0.0001 ppm
Broad.Factor 0.2864 Hz
Window Exponential
RGain 56

Operator__________

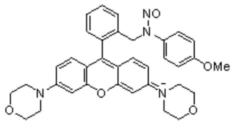

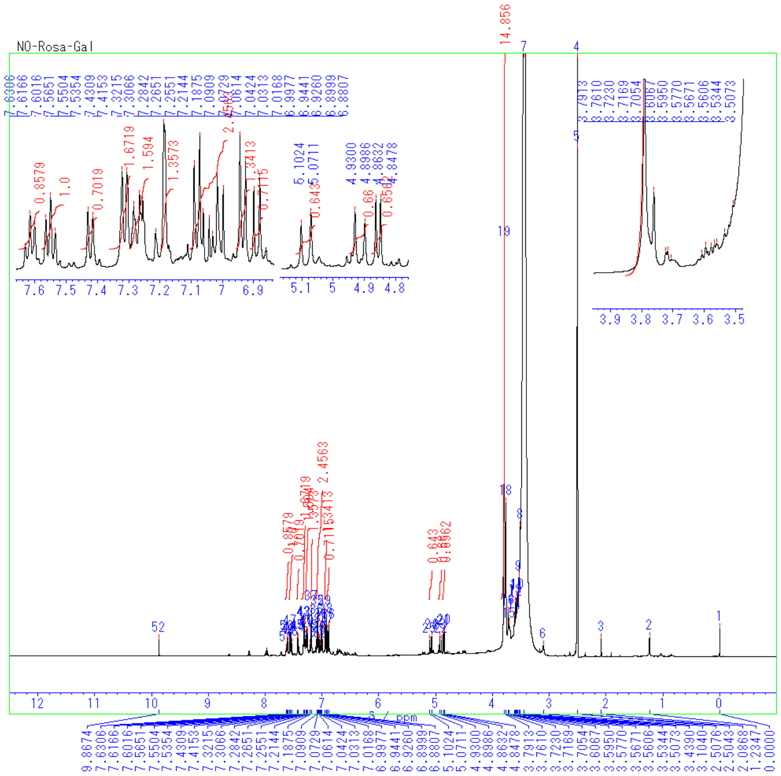

Date 07/Dec/2017 16:23:40
Comment: NO-Rosa-Gal
ObsNuc ¹H
ExMode PROTON
ObsFreq 499.92 MHz
ObsSet 0.0 kHz
ObsFine 8.0297 Hz
Point 131072(ZeroFill:×4)
Frequecy(Span) 8012.82 Hz
Scan 16
AcqTime 4.0894 s
PD 1.5 s
Pulse1 3.85 µs
IrrNuc ¹³C
ProbeHeaD
Instrument Varian UNITY
Pulse Program
Gradient Program
Temperature 25.0 ℃
Solvent dmso
Reference 0.0 ppm
Broad.Factor 0.0306 Hz
Window Exponential
RGain 34

Operator__________

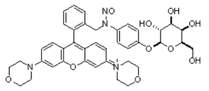

C. Absorption spectra

Absorption spectra of the compounds (10 μM) in 100 mM HEPES buffer (pH 7.3, DMSO 0.1%) were recorded on Agilent 8453 (Agilent Technologies).

D. Fluorescence spectra

Fluorescence spectra of the compounds (10 μM) in 100 mM HEPES buffer (pH 7.3, DMSO 0.1%) were recorded on RF-5300 (Shimadzu). Excitation wavelength was 510 nm. Each slit width was set at 3 nm.

E. Fluorescence quantum yield

Relative fluorescence quantum yield was obtained by comparing the area under the emission spectrum of the sample solution in 100 mM HEPES buffer (pH 7.3, DMSO 0.1%) with that of rhodamine B in EtOH, whose quantum yield is 0.49.

F. Fluorescence lifetime

The fluorescence lifetime of each compound (10 μM) in 100 mM HEPES buffer (pH 7.3, DMSO 0.1%) was measured with a time-correlated single-photon counting fluorometer (Hamamatsu, Quantaurus-Tau C11367). Samples were excited using a picosecond pulsed laser diode (Hamamatsu, PLP-10, excitation wavelength; 481 nm, pulse width; 80 ps, repetition rate; 1 MHz). Fluorescence decay curves were measured three times and analyzed by deconvolution.

G. NO measurement with an NO electrode

NO release was measured with an NO electrode, ISO-NOP (World Precision Instruments), and recorded on LabChart7 (ADInstruments). Light irradiation was conducted with a MAX-303 (Asahi Spectra) equipped with 530–590 nm band-pass filter (light intensity: 70 or 120 mW/cm^2).

H. NO release quantum yield

Measurement of the amount of NO released during irradiation: A solution of NO-Rosa5 (10 μM) in 100 mM HEPES buffer (pH 7.3, total volume: 3 mL) containing 0.1% DMSO was placed in a quartz cell and irradiated at 510 nm (band width was 10 nm) for 1 min with the Xe lamp of a fluorescence spectrometer, RF5300 (Shimadzu). The amount of NO released was measured with an ISO-NOP (World Precision Instruments) and recorded on LabChart7 (ADInstruments).
Measurement of the amount of photons: Potassium ferrioxalate (200 mg) was dissolved in 5 mL of 0.05 M H_2SO_4. The solution (3 mL) was irradiated under the same conditions as mentioned above. After irradiation, an aliquot (500 μL) was mixed with 500 μL of a buffer solution containing phenanthroline (2.25 g of NaOAc, and 10 mg of phenanthroline were dissolved in 10 mL of 0.5 M

H_2SO_4). The absorption spectrum of the mixture was recorded on an Agilent 8453 spectrometer. The absorption spectrum of the non-irradiated mixture was also recorded. The photon number was calculated from the absorption difference.

I. Electrochemical analysis

Cyclic and differential pulse voltammetry (CV and DPV) were performed on an ALS Electrochemical Analyzer (ALS-1140A) using Pt wire electrodes under an argon atmosphere. Rosa-Mor or *N*-methyl-*N*-nitrosoaminophenol (S26) was dissolved in MeCN (100 μM) containing *n*-$Bu_4N \cdot BF_4$ (0.1 M) as supporting electrolyte. CV or DPV experiments were run at ca. –30 ºC on a MeOH/liq. N_2 bath. Ferrocene, whose half-wave potential in the ferrocene/ferrocenium redox couple (Fc/Fc^+) is 0.420 V/SCE, was used as a standard.

J. Photodecomposition detection using LC-MS

A sample solution (total volume 10 mL) of NO-Rosa5 or NO-Rosa6 (10 μM) in 100 mM HEPES buffer (pH 7.3, DMSO 0.1%) was irradiated with MAX-303 (77 mW/cm^2, 530–590 nm). An aliquot of the solution (20 μL) was loaded onto an GL Science Inertsil ODS3 column (5 μm; 2.1×150 mm) fitted on a Waters ACQUITY/Quattro Premier XE system. MilliQ containing 0.1% formic acid (A) and MeCN containing 0.1% formic acid (B) were used as developing solvents. Gradient conditions were as follows: 0 min, A 95% and B 5% → 2 min, A 95% and B 5% → 3 min, A 90% and B 10% → 15 min, A 20% and B 80% → 17 min, A 20% and B 80% → 18 min, A 0% and B 100% → 25 min, A 0% and B 100% → 26 min, A 95% and B 5% → 30 min, A 95% and B 5%; ion mode, positive.

K. Tracking enzymatic hydrolysis of NO-Rosa-Gal using HPLC

A sample solution (total volume 10 mL) of NO-Rosa-Gal (10 μM), and β-D-galactosidase (10 U/mL) in HBSS was incubated at 37 ºC. An aliquot of each solution (20 μL) was loaded onto a Shenshu Pak C18 column (5 μm; 150 × 4.6 mm) fitted on a Shimadzu HPLC system, and the eluates were monitored with a photodiode array detector. MilliQ water containing 0.1% TFA (A) and MeCN containing 0.1% TFA (B) were used as developing solvents. Gradient conditions were as follows: 0 min, A 80% and B 20% → 20 min, A 20% and B 80%.

L. Light and enzyme specific NO release in cellular condition

LacZ (+) or *LacZ* (–) HEK293 cells were plated on 3.5 cm glass dishes at 2.0×10^5 cells/dish with 2 mL of Dulbecco's Modified Eagle Medium containing Mg^{2+} and Ca^{2+} (DMEM). The cells were incubated at 37 °C in a humidified atmosphere of 5% (v/v) CO_2 in air for 2 days. The cells were washed with Dulbecco's Phosphate-Buffered Saline (D-PBS, 2 mL×3). The cells were incubated with 10 μM DAF-FM DA (DMSO 0.2%) for 30 min under the above conditions. Then, the cells were

washed with D-PBS (2 mL×3), and the cells treated with 10 μM NO-Rosa-Gal (DMSO 0.1%) for 1 hr under the above conditions. The cells were irradiated with a MAX-303 (530−590 nm, 84 mW/cm^2) for 15 min. Before and after irradiation, the cells were examined under a confocal fluorescence microscope (Olympus, IX71).

3. Figures, table and Scheme for supporting data

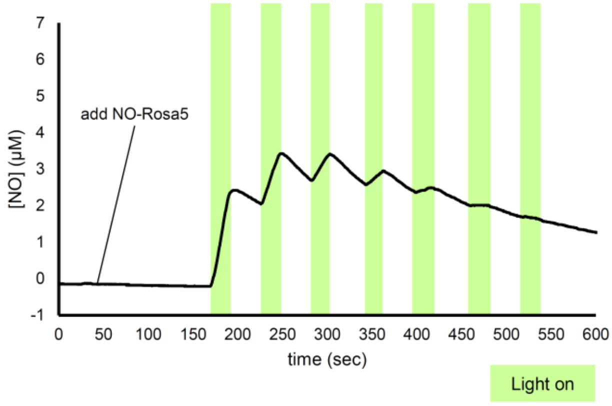

Figure S1 Temporal control of NO release from NO-Rosa5. A solution of NO-Rosa5 (10 μM) in HEPES buffer (100 mM, pH 7.3, DMSO 0.1%) was irradiated with a MAX-303 (Asahi Spectra) fitted with a 530–590 nm band-pass filter (light intensity: 70 mW/cm^2). The amount of NO was monitored with an NO electrode, ISO-NOP (World Precision Instruments) and recorded on LabChart7 (ADInstruments).

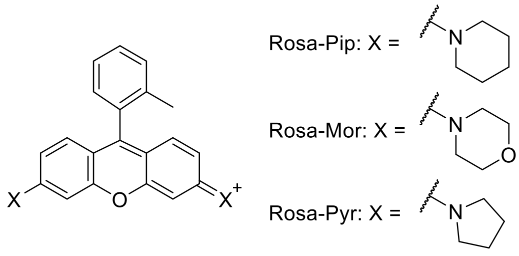

Figure S2 Structures of reference compounds

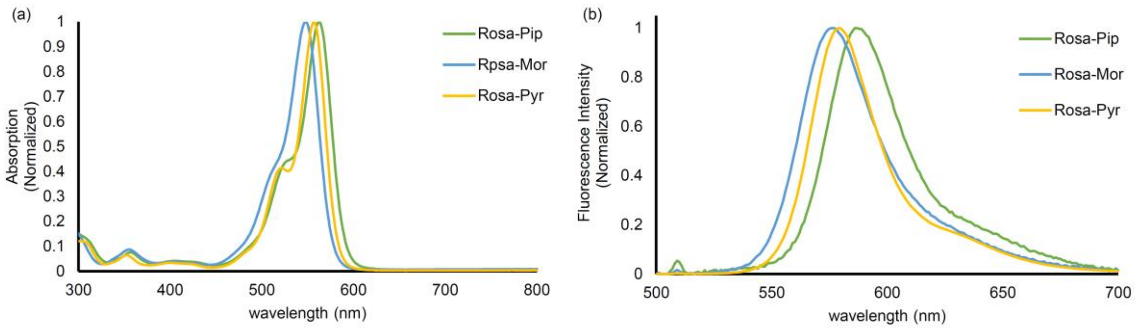

	Rosa-Pip	Rpsa-Mor	Rosa-Pyr
λ_{max}	562	548	557
ε_{max}	1.25×10^5	1.14×10^5	1.53×10^5

	Rosa-Pip	Rosa-Mor	Rosa-Pyr
λ_{max}	587	576	579
Φ_F	0.12	0.16	≈1

Figure S3 Absorption (a) and fluorescence (b) spectra of reference compounds in HEPES buffer (100 mM, pH 7.3, DMSO 0.1%).

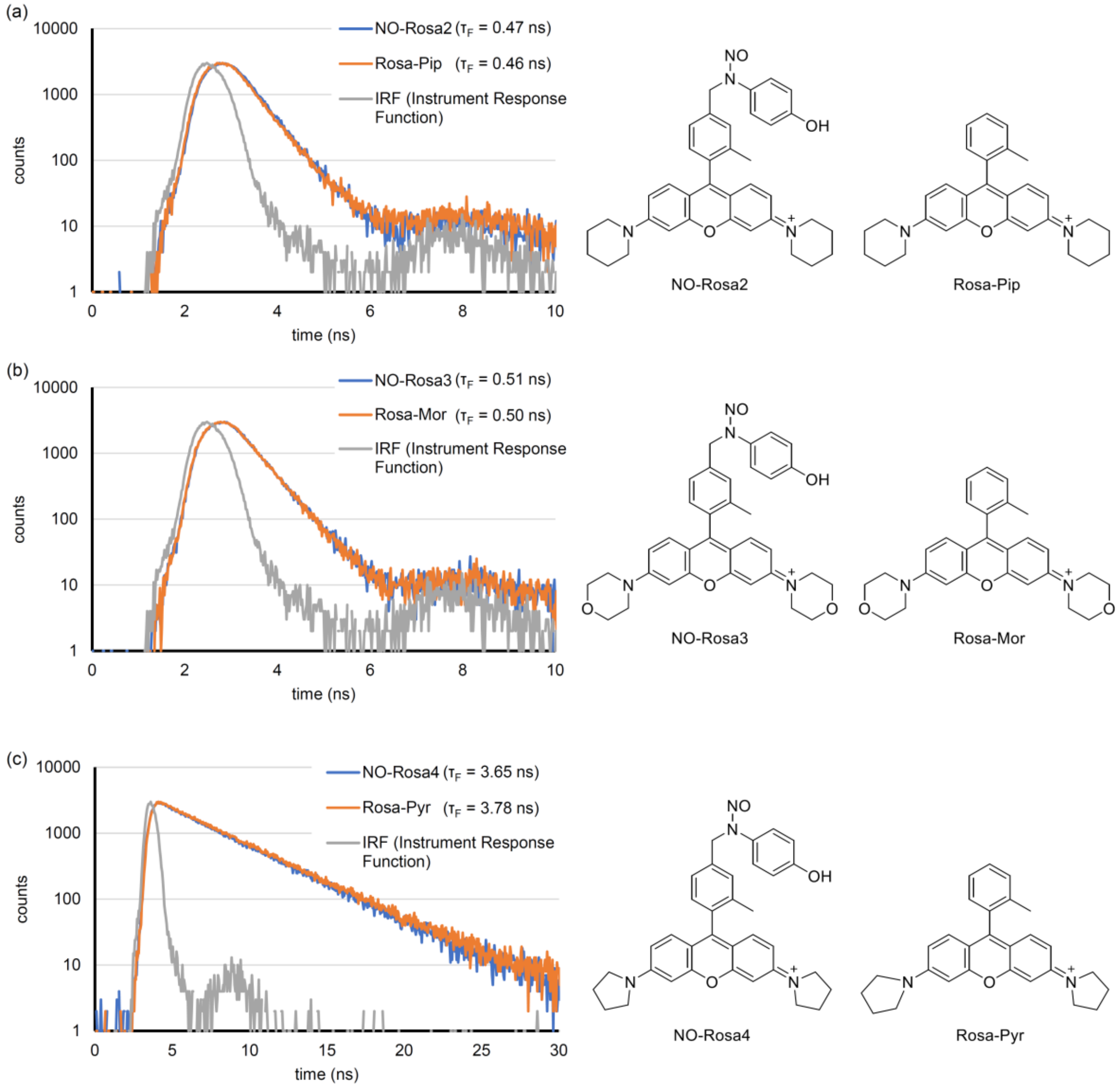

Figure S4 Fluorescence lifetime measurement: (a) NO-Rosa2 and Rosa-Pip; (b) NO-Rosa3 and Rosa-Mor; (c) NO-Rosa4 and Rosa-Pyr.

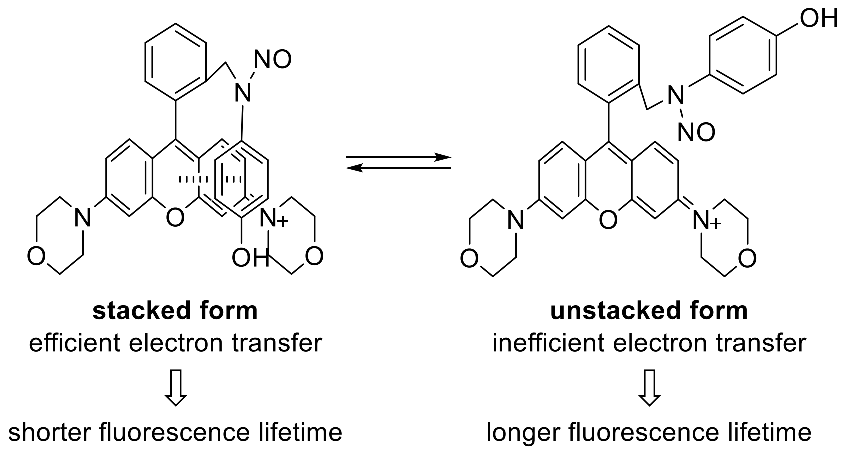

Figure S5 Plausible conformational isomers of NO-Rosa5 in its excited state

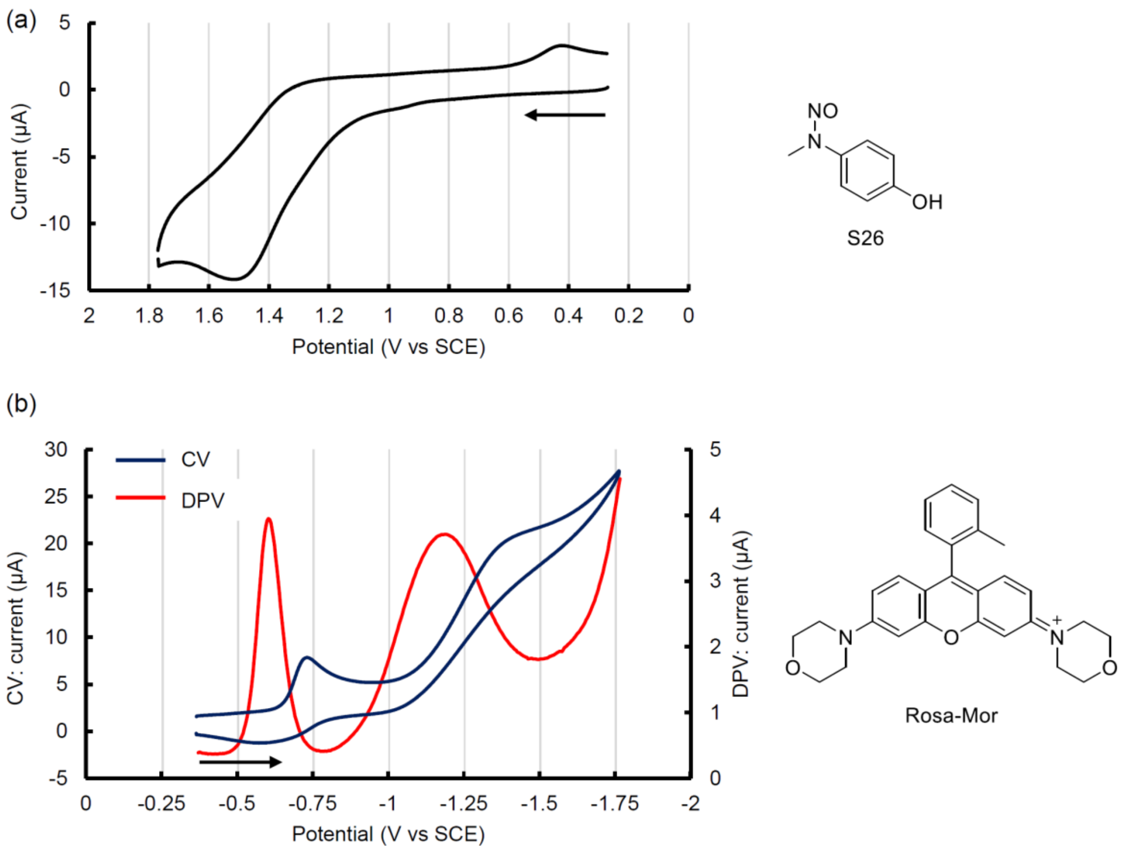

Figure S6 Cyclic voltammogram of S26 (100 μM) (a); cyclic and differential pulse voltammograms of Rosa-Mor (100 μM) (b) in MeCN/[n-Bu_4N][BF_4] (0.1 M).

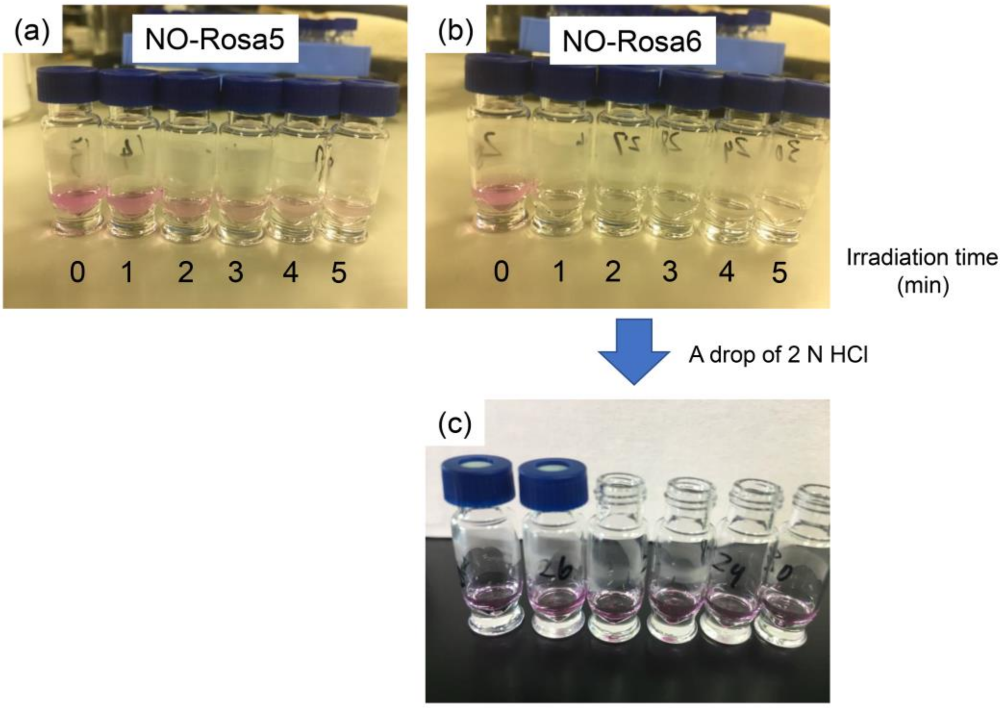

Figure S7 Color change of irradiated solutions of NO-Rosa5 and NO-Rosa6. (a) A solution of NO-Rosa5 after 0, 1, 2, 3, 4, and 5 min irradiation; (b) A solution of NO-Rosa6 after 0, 1, 2, 3, 4, and 5 min irradiation; (c) To each solution in (b) was added a drop of 2 N HCl.

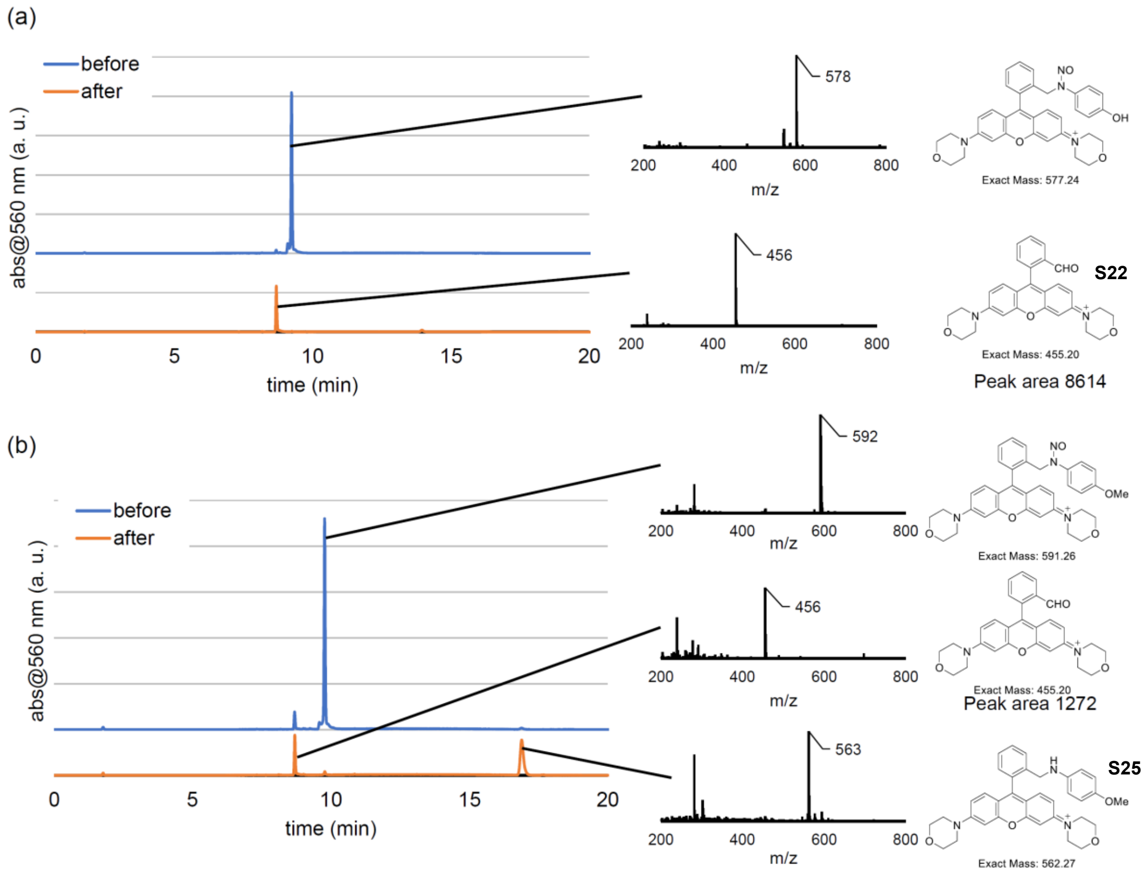

Figure S8 LC-MS analysis of photodecomposition. (a) A chromatogram of a solution of NO-Rosa5 before (blue line) and after (orange color line) irradiation; (b) A chromatogram of a solution of NO-Rosa6 before (blue line) and after (orange color line) irradiation.

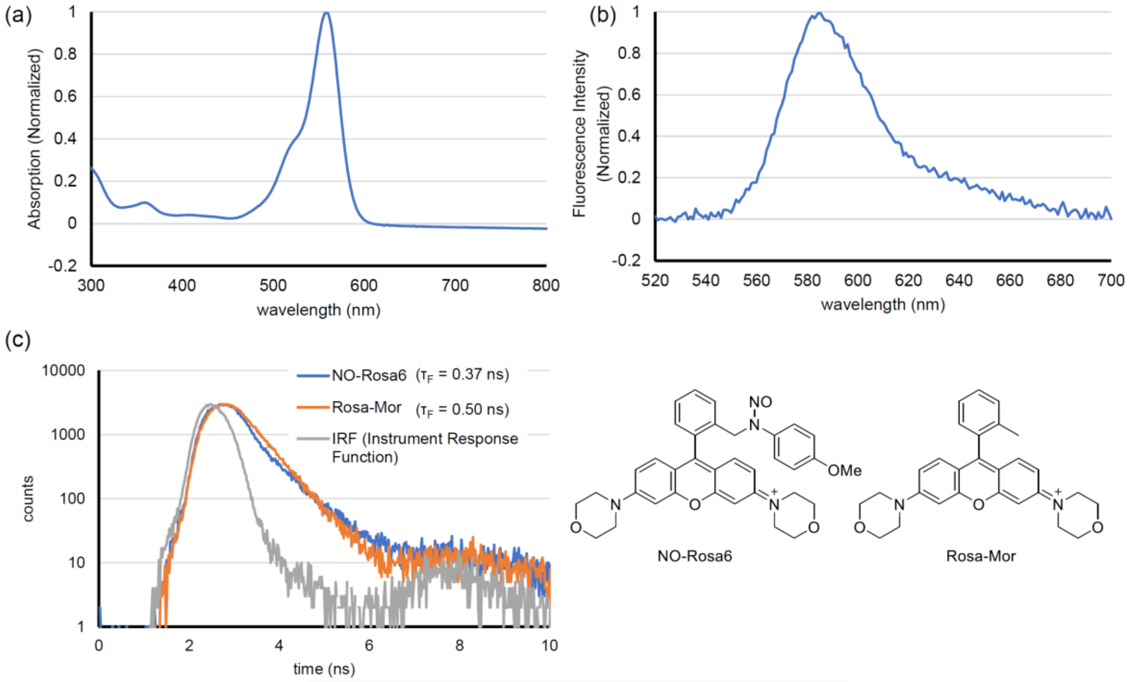

		NO-Rosa6
Absorption	λ_{max}	558
	ε_{max}	3.77×10^4
Fluorescence	λ_{max}	585
	Φ_F	0.05

Figure S9 Absorption (a), fluorescence (b) spectra, and time-resonance fluorescence plot (c) of NO-Rosa6.

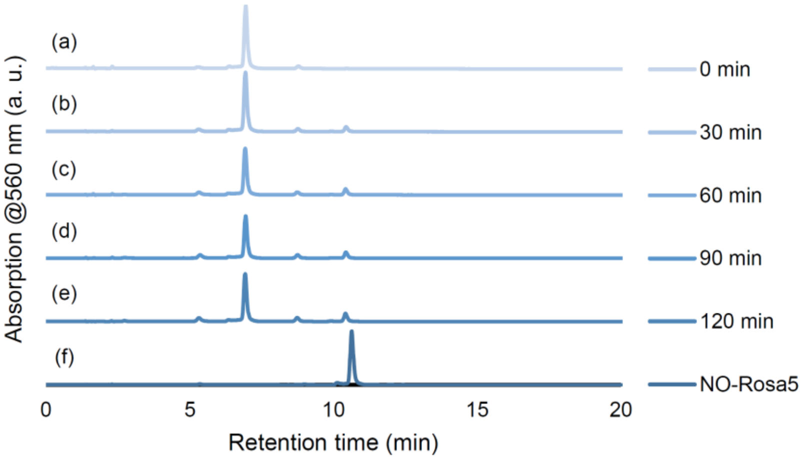

Figure S10 Non-enzymatic hydrolysis of NO-Rosa-Gal monitored by HPLC. A solution of NO-Rosa-Gal (10 μM) in HBSS (0.1% DMSO) was incubated in the absence of β-galactosidase at 37 ºC for 0 min (a), 30 min (b), 60 min (c), 90 min (d), and 120 min (e). The bottom chromatogram (f) is that of authentic NO-Rosa5 (10 μM).

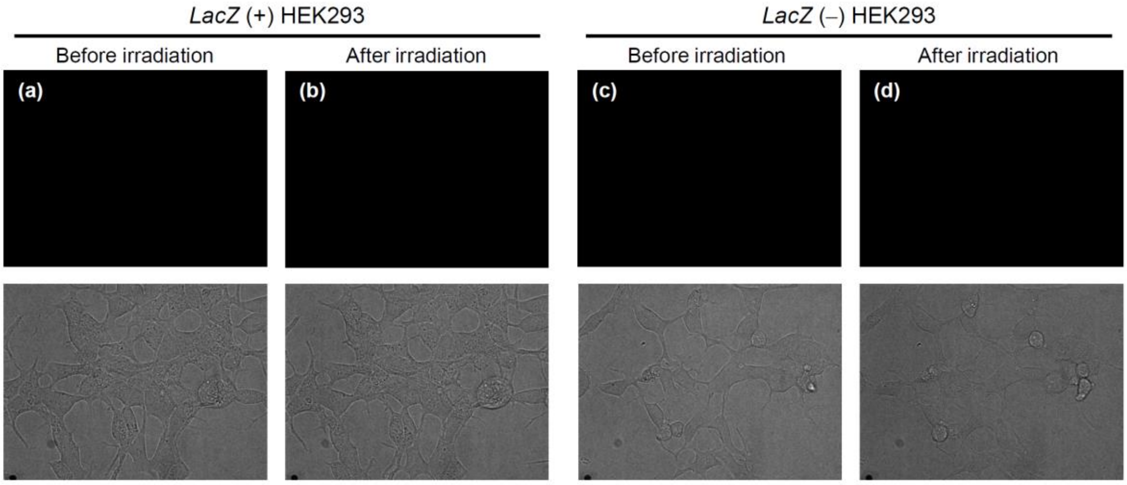

Figure S11 Fluorescence imaging of NO release in *lacZ*-HEK293 cells using DAF-FM DA. Cultured HEK293 cells were treated with vehicle (DMSO) and NO-Rosa-Gal (10 μM). The dishes were then photoirradiated with blue light (530−590 nm, 84 mW/cm^2 for 15 min). The dishes were observed with a confocal fluorescence microscope. Upper figures are green fluorescence, and bottom figures are bright filed. (a) before photoirradiation to *lacZ* (+) cells, (b) after photoirradiation to *lacZ* (+) cells, (c) before photoirradiation to *lacZ* (-) cells, (d) after photoirradiation to *lacZ* (-) cells, Upper: fluorescence images. Lower: bright filed images.

Table S1 Fluorescence lifetimes and pre-exponential factors of NO-Rosa5 (10 μM) in 100 mM HEPES buffer (pH 7.3, DMSO 0.1%).

Compound	τ_{F1} / ns	τ_{F2} / ns	A_1 / %	A_2 / %	$<\tau_F>$ / ns
NO-Rosa5	0.32	1.0	94	6	0.36

The amplitude-averaged lifetime ($<\tau_F>$) was calculated according to the following equation,

$$<\tau_F> = A_1\tau_{F1} + A_2\tau_{F2}$$

where τ_{F1}, τ_{F2}, and A_1, A_2 are the fluorescence lifetimes and the pre-exponential factors, respectively.

Scheme S1 Synthesis of NO-Rosa1

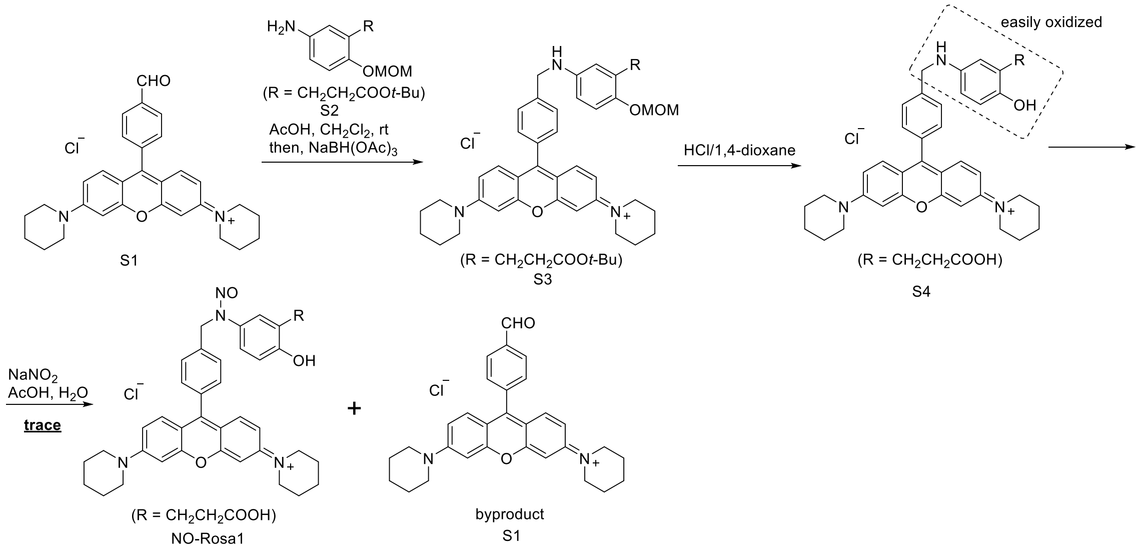

Scheme S2 Synthesis of NO-Rosa2–4

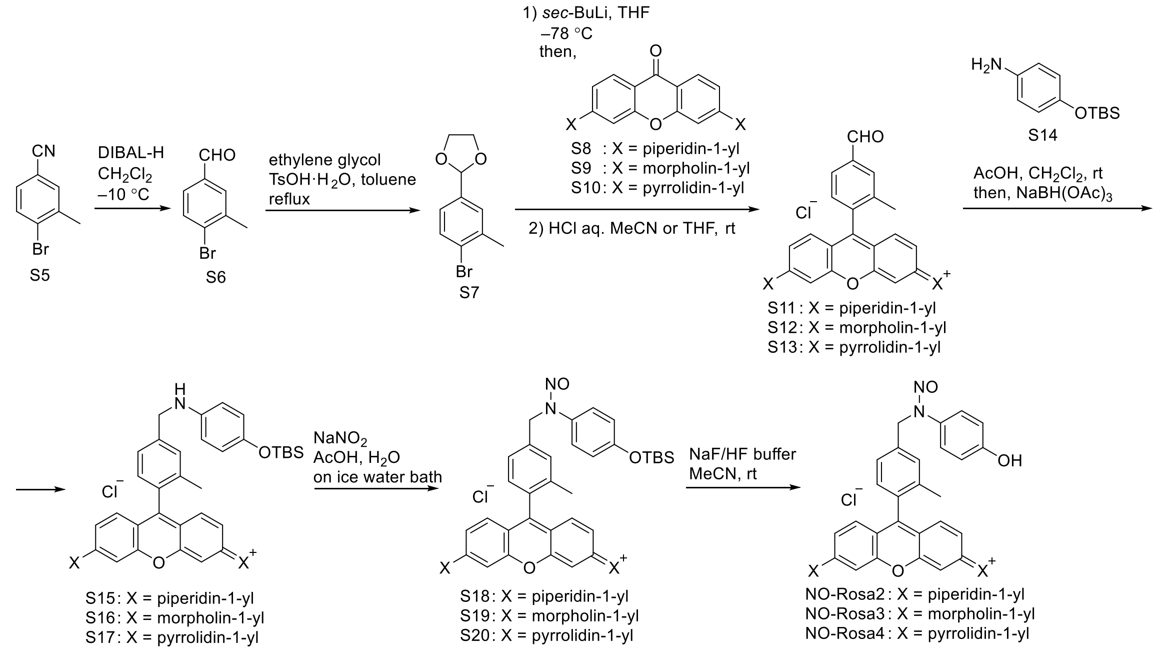

Scheme S3 Synthesis of NO-Rosa5

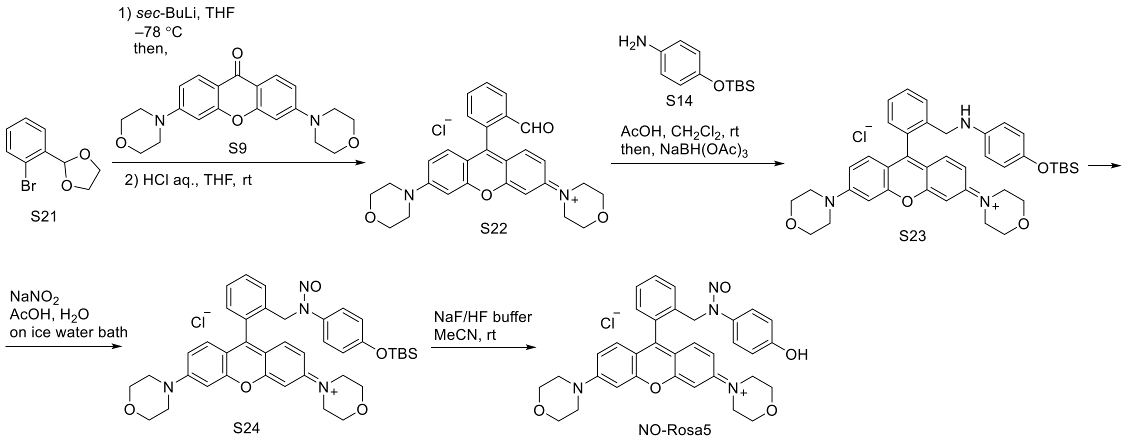

Scheme S4 Synthesis of NO-Rosa6

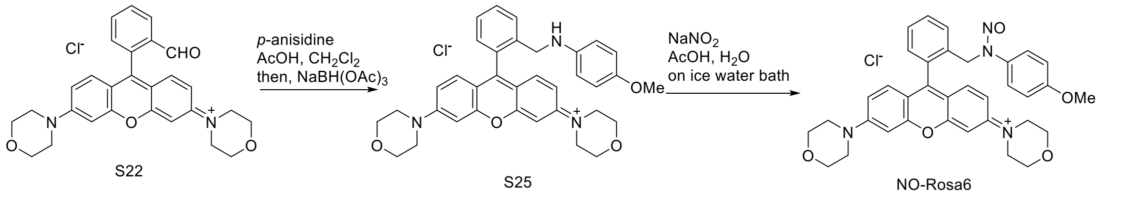

Scheme S5 A plausible reaction mechanism of NO-Rosa6 under photoirradiation

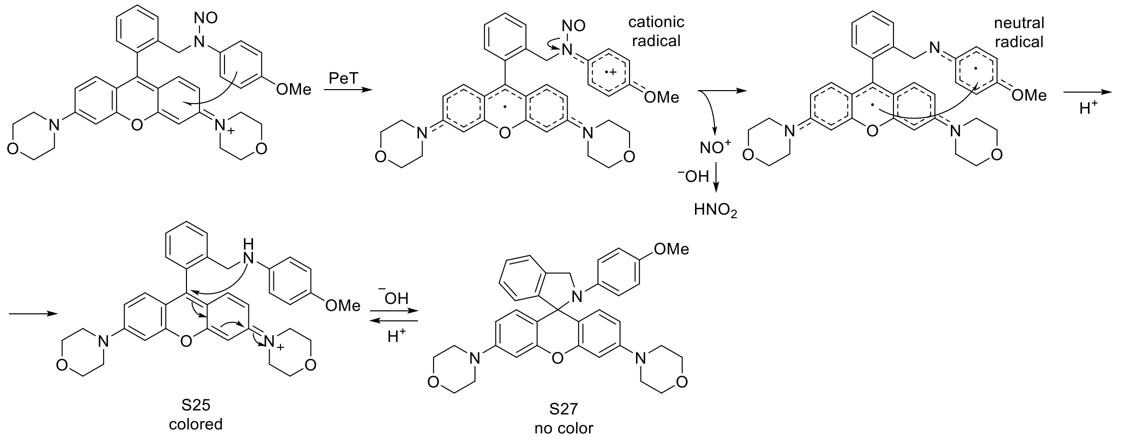

Scheme S6 Synthesis of NO-Rosa-Gal

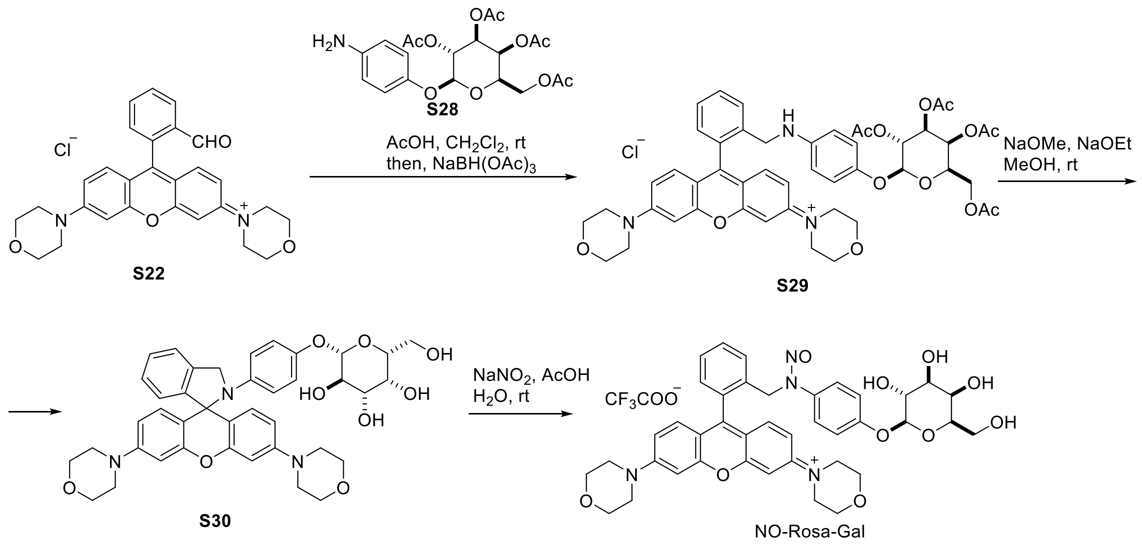

Supplement: Supplementary file 1 — Supplementary Information [file 41598_2018_38252_MOESM1_ESM.pdf]
